# When repeated presentation of visual feature bindings does and does not result in learning: Visual short-term and long-term memory are distinct but work in tandem

**DOI:** 10.3758/s13421-025-01816-8

**Published:** 2025-11-29

**Authors:** Chaoxiong Ye, Qiang Liu, Robert H. Logie

**Affiliations:** 1 School of Education, Anyang Normal University, Anyang, China; 2 Department of Psychology, University of Jyvaskyla, Jyvaskyla, Finland; 3 Institute of Brain and Psychological Sciences, Sichuan Normal University, Chengdu, China; 4 Department of Psychology, University of Edinburgh, Edinburgh, UK

**Keywords:** Visual working memory, Visual array repetition, Feature binding, Study–test interval, No learning from repetition

## Abstract

Two experiments explored a previous finding that 120 repetitions of the same six-item array for change detection resulted in no or very slow learning. This contrasts with learning from six repetitions tested by recall. Shimi and Logie (*Quarterly Journal of Experimental Psychology, 72,* 1387–1400, 2019) proposed that array repetition for change detection is supported by a limited capacity, temporary visual cache memory, with contents replaced by the next study array, even if it is identical. This is coupled with a weak episodic trace that strengthens across repeated presentations but requires more than 60 repetitions for learning. Experiment 1 tested whether (a) a short study–test interval would result in reliance on the visual cache with no evidence of learning across 120 repetitions, and (b) a longer study–test interval would gradually strengthen the episodic trace but require many repetitions for learning. A 500-ms study–test interval showed no learning after 120 repetitions, and participants reported being unaware of the repetition. A 5,000-ms study–test interval showed performance improvements, but only after 40 repetitions, and participants reported becoming aware of the repetition. In Experiment 2, different arrays on each of 120 trials with short and long study–test intervals showed the same lack of learning found for the 500-ms study–test interval in Experiment 1. Results appear consistent with a limited capacity visual cache memory for change detection that retains the array only for the current trial, working in tandem with a weak episodic trace that accumulates across trials but only supports performance after multiple repetitions and longer study–test intervals.

**Supplementary Information:**

The online version contains supplementary material available at 10.3758/s13421-025-01816-8.

## Introduction

When moving through our environment, often there are rapid changes in the visual information that should be observed and retained only briefly with constant updating for successful and safe interaction with the world. This ability to keep track of rapid changes around us often is discussed as a key function of working memory (see reviews in R. H. Logie, Camos et al., 2021). In contrast, there are combinations of shape and color that tend to be consistent and repeated, and learning these consistencies from repeated viewing can be important for daily living. From an evolutionary perspective, early humans and mobile, nonhuman animals might have developed something like a visual working memory (sometimes also referred to as visual short-term memory) for survival, such as for keeping track of the current and rapidly changing position of a predator, or of a potential meal for the individual or their community (e.g., Wynn, 2002; Wynn & Coolidge, 2010). Viewing of the target might be fragmentary and intermittent, so there is reliance on rapid updating of the current visual representation. However, it would also have been crucial to learn, from repeated visual and other experiences, how to identify a predator or prey as well as other consistencies in the environment. In the contemporary scenario of a busy freeway, the driver has to keep track of the current and rapidly changing positions of nearby traffic, not all of which is in current view. There could be major disadvantages to basing current action on memory for a black Ferrari that passed by 10 minutes ago when now there is a large truck behind us. However, if that same Ferrari is seen multiple times, more details may be added to a more enduring representation of its characteristics. In the present experiments, we investigated conditions under which multiple repeated presentations of the same stimulus array leads to a complete lack of learning in feature change detection, and conditions that do lead to learning from repeated presentation.

Multiple studies have demonstrated that repeated presentation of the same list of numbers or words for immediate recall, interspersed with novel lists, results in gradual improvement in recall of the repeated list even when participants appear unaware of the repetition. This is in contrast to no improvements in recall across trials with lists that are novel on each trial or that are interspersed between trials with the repeated item (e.g., Araya et al., 2024; Caird, 1964; Cunningham et al., 1984; Hebb, 1961; Hitch et al., 2005; Musfeld et al., 2023; Oberauer et al., 2015; Page et al., 2006, 2013). A few studies have used this paradigm to investigate learning of sequences of nonverbal items presented visually (e.g., Couture & Tremblay, 2006; Gagnon et al., 2004, 2005; Musfeld et al., 2024; Page et al., 2006; Sukegawa et al., 2019; Turcotte et al., 2005). As with verbal sequences, all of these studies have shown that when the same sequence of pictures or order of appearance of dots is shown repeatedly on some of the trials, participants show improvement in the recall of the repeated sequence compared with the novel sequences on the interpolated trials. This kind of evidence suggests that, although participants expect a series of novel items, and might or might not be aware of repeated stimuli, nevertheless they retain a trace of each item after every trial, allowing a strengthening of the memory trace every time a particular item is repeated. Musfeld et al. (2023) have suggested that this gradual strengthening may lead to awareness of the repetition by individual participants who then show evidence of learning, and participants show no learning until they become aware of the repetition. As more participants become aware of the repetition, performance improves in the group aggregate data. That is, awareness of the repetition precedes improvements in performance as more repetitions of the target stimulus occur.

In contrast, several studies have shown that repeated presentation of exactly the same visual stimulus fails to lead to learning or improved memory performance. Nickerson and Adams (1979) demonstrated that a sample of US residents was unable to recall the visual details on a 1 cent coin, or even to recognize the correct details among several modified versions. This was despite having seen and used one cent coins many thousands of times in their lives. Richardson (1992) reported a similar lack of learning of details on British coins and postage stamps among people who had lived in the UK for most or all of their lives. Bekerian and Baddeley (1980) reported a complete lack of learning of changes to sound broadcasting by the BBC at a time when this relied on tuning in to radio frequencies. This was despite being informed of the changes on average 25 times per day over many weeks.

The lack of learning from repeated presentation of visual details has also been demonstrated in laboratory studies. For example, Olson and Jiang (2004) presented a study array with squares or shapes in random locations, followed by a test array of items, with one item highlighted. The participant had to indicate whether the highlighted item had been shown in one of the locations in the previous array. Unknown to the participants, six specific configurations of items were each repeated across 24 trial blocks, interspersed with trials on which the spatial locations of the items in the study array varied randomly. Despite some of the configurations being seen repeatedly 24 times, recognition performance on the repeated arrays was no better than for the randomly changing arrays.

R. H. Logie et al. (2009) showed participants arrays of six different colored shapes, and they were required to remember the specific combination (binding) of features (color, shape, and location) for each item in the study array shown on each trial. Following presentation of the study array, a test array depicted the same six colors, six shapes, and six locations as before. Participants had to decide whether or not two of the shapes had swapped colors (25% trials), or two colors had swapped shapes (25% trials). This was a test of recognition memory known as change detection among the feature bindings for the previously presented study array (e.g. Gajewski & Brockmole, 2006; Schneegans & Bays, 2019; Wheeler & Treisman, 2002). On the remaining 50% of trials in R. H. Logie et al. (2009), the study and test arrays were identical in shape, color, and location. In one experiment, exactly the same study array was shown on every third trial across 72 trials, so repeated 24 times. All other trials showed novel combinations of shape, color, and location. Participants were required throughout the trial to repeat orally an irrelevant word (articulatory suppression) to mitigate use of verbal labels for the stimuli. There was no difference in performance for repeated and nonrepeated trials, that is there was no evidence of learning from multiple repetitions of the same stimulus array. To investigate whether 24 repetitions might have been insufficient to detect learning, or that the novel arrays disrupted memory for the repeated array, in Experiment 2 exactly the same study array (identical combinations of shape, color and location) was shown across 60 trials, with no novel arrays shown. On half of the trials the test array was identical to the study array. On the remaining trials, the test array showed a swap of colors between two shapes, or a swap of shapes between two colors. That is, participants viewed exactly the same array a total of 90 times (60 study arrays plus 30 no-change test arrays) and yet showed no evidence of improving their performance across trials. These results confirmed that the findings by Olson and Jiang (2004) for monochrome squares and monochrome shape arrays generalise to arrays of colored shapes. The results also suggest that the lack of improvement with repetition in the earlier study was not simply due to Olson and Jiang having too few repetitions (24) of each target array, or because of visual interference from presentation of interspersed novel arrays.

Crucially, in R. H. Logie et al.’s (2009) Experiment 3, instead of a test array, a single test probe was presented in a location that was previously occupied by one of the items in the study array, and participants reported the color and shape names of the probed item. Testing memory with probed oral recall resulted in rapid improvement in recall performance across repeated presentations of the same stimulus array. That is, performance improvements resulted from repeated oral recall of color–shape–location combinations across trials, not from repeated presentation and recognition of the same array. Previous studies, cited above, that consistently showed learning from repetition, used recall of the verbal or visual material (e.g., Araya et al., 2024; Hebb, 1961; Page et al., 2013; Sukegawa et al., 2019). Therefore, the contrasting results across studies appear to arise from the use of recall that results in clear evidence of performance improvements from repeated use of the same stimuli, compared with the use of recognition that results in no performance improvements even when a stimulus array is viewed as many as 90 times.

Shimi and Logie (2019, Experiment 1) again adopted change detection with a repeated array, but presented exactly the same study array (same combinations of color, shape, and location) 120 times, double that in R. H. Logie et al. (2009), with arrays of four or of six colored shapes. Half of the test arrays were identical to the repeated study array, so participants viewed exactly the same array 180 times (120 times as study array and 60 times as the no-change test array). For the other half of the test arrays, two of the shapes swapped colors, or two of the colors swapped shapes, but the other items were the same as in the repeated study array. With the repeated four-item array performance for all participants was above 90% within the first few trials, with some participants quickly reaching 100%. With the repeated six-item array, the group data showed clear evidence that they remembered four of the six items on the first trial block of 20 trials, and for multiple trials thereafter. There was subsequent performance improvement, but only after around 60 trials (90 repeated presentations) followed by further, but slow improvements across the remaining 60 trials. This is consistent with the lack of learning in R. H. Logie et al. (2009) across 60 repetition trials. In addition, participants were asked after the experiment whether they had been aware that the same array had been repeated. The participants who reported becoming aware of the repetition showed clear improvements in change detection performance but only after around 60 trials, and it was primarily data from these participants that resulted in improvements in overall group means. These participants continued to improve, but even after 120 trials, performance was not consistently at ceiling. The remaining participants who reported no awareness of the repetition began to show performance improvements after around 100 trials, but reached only around 80% performance after 120 trials with exactly the same six-item array. In a second experiment Shimi and Logie (2019) used visual reconstruction of the arrays instead of change detection, and found very clear and rapid performance improvements within the first 20 trials for all participants. Thus, as for R. H. Logie et al. (2009), recall of six-item arrays resulted in rapid learning, whereas change detection resulted in little to no, or very slow learning after more than 60 repeated presentations of exactly the same array, with more rapid learning observed on the later trials among participants who reported that they had become aware of the repetition.

Shimi and Logie (2019) suggested that performance on each trial for change detection relies on a temporary visual short-term memory system, or a visual cache (R. H. Logie, 1995, 2003, 2011, 2023), with a capacity to store around four items at a high level of precision (e.g., Cowan, 2001; Luck & Vogel, 1997; Schneegans & Bays, 2019). The finding that performance with a four-item array was close to ceiling before any learning could have occurred, is consistent with this interpretation. Performance with the six-item arrays across 40–60 trials was around 70%, suggesting that participants successfully recalled four of the six items on any one trial, and continued to recall only four items on every trial, even after an identical study array had been presented on every trial. This is consistent with the hypothesis that participants relied on a limited capacity visual cache, distinct from episodic long-term memory, with its contents overwritten by the array on the next trial even if the next array was identical. There was therefore no residual trace in the cache from trial to trial to support learning from repeated presentation.

One alternative theoretical framework known as “embedded processes” (e.g., Cowan, 1999, 2023; Cowan et al., 2021) assumes that the current contents of working memory comprise currently activated traces from long-term memory, coupled with a limited focus of attention at any one time on a small amount of the activated traces. On this basis, repeated presentation of the same combination of stimulus features should result in repeated activation of the same feature combinations which are then repeatedly in the focus of attention. This should result in strengthening of the activated traces and performance improvements across repetitions showing evidence of learning. The complete lack of performance improvement across as many as 60 repeated presentations of the same array, and after even more repetitions for some participants would be difficult to explain within this framework without additional assumptions.

A possible additional assumption is that there are ‘peripheral components’ of working memory, one of which can store the phonological codes of a small amount of verbal information in a passive form and separately from episodic long-term memory (Barrouillet & Camos, 2021; Cowan et al., 2014). This is compatible with the concept of the phonological loop proposed by Baddeley (1986; Baddeley & Hitch, 1974). Barrouillet and Camos (2021) have suggested that there is also a peripheral component that can store a small amount of visual information. This latter proposal is consistent with the concept of a passive and limited capacity temporary visual short-term memory or visual cache (R. H. Logie, 1995, 2003, 2011; R. H. Logie, Belletier et al., 2021; Phillips & Christie, 1977), that can store a small amount of information for the duration of a single trial separately from episodic memory, with its contents replaced a few seconds later by the stimulus on the next trial. The evidence from Olson and Jiang (2004), R. H. Logie et al. (2009), and Shimi and Logie (2019) suggest that the contents of the cache are replaced by the stimulus on the next trial even if that stimulus is identical.

The suggestion that performance on each trial is supported by a temporary visual cache with no residual trace that can support learning offers an explanation as to why there is no evidence of learning across 60 repetitions of the same stimulus array. However, this leaves the question of why any learning occurs at all with more than 60 repetitions and why some participants become aware of the repetition. Shimi and Logie (2019) suggested that, in addition to a temporary, limited capacity visual cache supporting performance on each individual trial, a weak episodic trace was also formed in episodic long-term memory on each trial, and the strength of this trace increased very slowly as the number of repetitions increased, but the episodic trace was initially too weak to support performance. Only after more than 60 repetitions was that trace strong enough to support learning and performance improvements in some participants and for some participants to become aware of the repetition. This would be compatible with Cowan’s embedded processes account if it is assumed that there is indeed repeated activation of long-term memory traces, but this is weak on any given trial and so it takes many repetitions for those traces to support performance. For some participants, after around 60 repetitions, the trace becomes sufficiently strong to supplement performance, so that the contents of the cache and the episodic trace could both contribute and performance increased as a result. With additional repetitions, the strength of the episodic trace increased further, so that very gradually performance became more driven by the strengthening episodic trace than by the limited capacity visual cache. For other participants, the strength of the episodic trace increased much more slowly, and required 100 or more repetition trials before supplementing performance, with some participants showing no performance improvements even after 120 repetitions.

A replication of the lack of recognition performance improvement with repeated presentation was reported by Souza and Oberauer (2022, Experiment 7) who, following Olson and Jiang (2004), presented colored square arrays for probed recognition with online data collection. The same array was displayed 24 times, with three trials showing novel arrays between each repeated trial. They offered a similar account to Shimi and Logie (2019), of contributions from a limited capacity working memory coupled with contributions from episodic memory when the episodic trace became sufficiently strong to be useful. This account also is consistent with the R. H. Logie (1995, 2003, 2011. 2018, 2023; see also Adam et al., 2024; Fukuda & Vogel, 2019; Hart et al., 2025) proposal that for any given cognitive task, there will be contributions to performance from multiple cognitive functions, including from a temporary visual cache and also, when memory traces are sufficiently strong, from episodic and semantic memory.

In seven other experiments, Souza and Oberauer (2022) used probed recall procedures and demonstrated that giving participants more time to consolidate the study array resulted in faster learning (see also Li et al., 2020; Ricker & Hardman, 2017; Schurgin et al., 2020). However, they did not vary consolidation time with their recognition procedure. If the Shimi and Logie (2019) interpretation offers an adequate account of the recognition data, then if participants are given more time between the study and test array on each trial, this should allow more time for consolidation of an episodic trace of the target array, thereby accelerating strengthening of that trace and reducing the number of repeated trials before performance starts to improve. Conversely, a shorter study–test interval might slow learning more than was found in those previous studies, or show no learning at all even with a large number of repetitions. This hypothesis was tested in Experiment 1 by varying the study–test interval with a repeated array on every trial and change detection between the study and the test array. Shimi and Logie (2019) and R. H. Logie et al. (2009) displayed the study array for 200 ms with a study–test interval of 2,000 ms. Experiment 1 had a shorter (500 ms), longer (5,000 ms), and the same (2,000 ms) study–test interval. Otherwise the procedure was the same as Shimi and Logie (2019) Experiment 1, with in-person testing in a lab.

## Experiment 1

### Method

#### Participants

Prior to data collection, we conducted an a priori power analysis for our 6 (blocks: 1 to 6) × 3 (study–test intervals: 500 ms vs. 2,000 ms vs. 5,000 ms) design, using a repeated-measures analysis of variance (ANOVA) as the primary analytical approach and focusing on the Block × Study–Test Interval interaction in accuracy. We anticipated a moderate-to-small effect size (e.g., η_p_^2^ ~ = 0.10, corresponding to *f* = 0.33) with a statistical power of 80% at a significance level of 0.05 (Faul et al., 2007). The power analysis was conducted using G*Power (Version 3.1.9.7), specifying an *F* test for a repeated-measures ANOVA with a within–between interaction, assuming three groups, six repeated measurements, a nonsphericity correction (ε) of 1, and a correlation among repeated measures of *r* =.5. Based on these parameters, the estimated minimum sample size was 36 participants.

Thirty-nine college students (31 women, eight men; *M* = 20.33, *SD* = 1.56) participated in Experiment 1 with monetary compensation. All participants reported having normal or corrected-to-normal vision and normal color vision, and provided written informed consent prior to participating in the study. Ethical approval for the study was obtained from the ethics committee of Sichuan Normal University. Participants were tested individually in a cognitive psychology lab.

#### Apparatus and stimuli

**Fig. 1:**
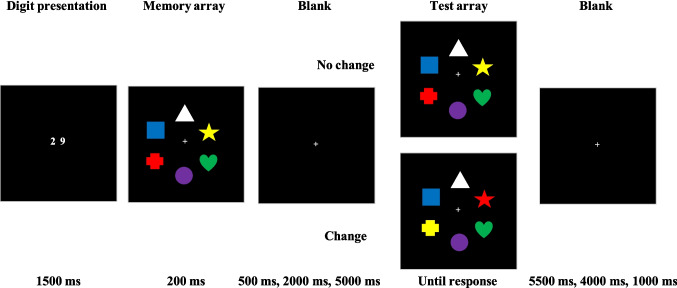
Illustration of a trial sequence for the change-detection task in Experiment 1. (Color figure online)

Experiment 1 followed a 6 (blocks: 1 to 6) × 3 (study–test intervals: 500 ms vs. 2,000 ms vs. 5,000 ms) repeated-measures factorial design, with block as a within-subjects factor, and study–test interval as a between-subjects factor balanced across participants (13 per group). This experiment mirrored the change-detection task in Shimi and Logie’s (2019) Experiment 1. Figure 1 illustrates task descriptions, trial sequences, and durations. On each trial, participants observed the study array of six colored squares for 200 ms, followed by a blank screen for 500 ms, 2,000 ms, or 5,000 ms, before the test array that remained visible until the participant responded. To equate total time (6,000 ms) for each trial across different study–test intervals, a blank screen of varying lengths was presented following test array removal: 5,500 ms for a 500-ms interval, 4,000 ms for a 2,000-ms interval task, and 1,000 ms for a 5,000-ms interval. During presentation of the test array, participants were instructed to indicate, via keyboard buttons (“j” for change, “f” for no change), whether or not a change had occurred between the study and test array. Changes involved either a color swap (two objects exchanged colors while retaining shapes and locations) or a shape swap (two objects exchanged shapes while retaining colors and locations), with the other four color–shape–location combinations identical to the repeated study array. The study array was different for every participant,

but each participant saw their allocated array repeated throughout. That is, the precise combinations of color, shape, and location for each of the objects were identical for the study array on every trial, and for the test array on half of the trials. Participants were not informed of this repetition. At the beginning of each trial, two random digits (1–9) were displayed for 1,500 ms. These digits were to be repeated orally by the participant (articulatory suppression) at a rate of two per second throughout the trial and until test array response. Articulatory suppression was included to prevent participants from subvocally rehearsing the names of the colors, shapes, or locations of the objects in the array during study presentation or retention interval. The experimenter monitored participants to ensure compliance with articulatory suppression instructions. Participants were randomly assigned to one of the three study–test intervals (500 ms, 2,000 ms, or 5,000 ms).

Stimuli were presented against a black backdrop using E-Prime 1.0 software (Psychological Software Tools, Inc). The array to be repeatedly presented for each participant was randomly generated at the beginning of the task, and the array objects were created as random combinations of distinct features drawn from a list of eight geometric shapes (arrow, circle, cross, diamond, heart, square, star, and triangle) and eight colors (blue, cyan, green, grey, magenta, red, white, and yellow), with no repetitions of color or shape within the resulting six-item array. Each item subtended approximately 3.46° × 3.46° of visual angle. The items were symmetrically arranged in six locations around an imaginary circle, each at a 5.63° angular eccentricity from a central fixation point (that remained on the screen throughout the trial) from a viewing distance of about 70 cm.

Participants completed four practice trials with different arrays from the main experimental trials, followed by six test blocks of 20 trials each, totaling 120 experimental trials for each participant. From these, 60 (50%) were no-change trials and 60 (50%) were change trials (25% color change and 25% shape change). Change trials were randomly mixed with no-change trials, and changes were equally probable across all locations in the array. With 120 trials, the same array was repeated 180 times: 120 times during study and 60 times for test on no-change trials. The proportion of same/different trials remained constant across blocks.

#### Procedure

Participants were seated in front of a computer screen and provided with written and oral instructions. During the practice trials, visual feedback (correct, incorrect) was displayed after each trial, but no feedback was given during experimental trials. The arrays in practice trials differed from each other and were different from those for experimental trials. Self-paced breaks were incorporated between the blocks in each task. Upon completion of all trials, participants were asked whether they perceived the stimuli presented in the study array as being the same or different on each trial. Regardless of their answer, participants then rated confidence in their response on a scale from 1 to 9. Higher scores indicated greater confidence. If the participant responded that they had noticed that arrays were the same, they were asked to estimate the block (1–6) when they began to notice.

#### Statistical design and analyses

We conducted separate analyses for memory accuracy and response times. Participants were instructed to prioritize accuracy over speed. Therefore we did not exclude trials based on very slow or unrealistically fast response times. However, we also analyzed only trials with response times between 200 ms and 2,000 ms, reported in Supplementary Material, Fig. S1−2 and Table S1−2. The pattern of results was consistent with the full dataset, confirming that the inclusion of all trials did not bias the findings. Therefore we report analyses of memory performance based on the full set of trials, regardless of response time.

Because participants had a short rest in between each block of 20 trials, and to allow comparison with results from previous studies (R. H. Logie et al., 2009; Shimi & Logie, 2019), we analyzed data aggregated within each of six blocks of 20 trials.

To assess whether varying the study–test inerval had an impact on change detection with a repeated array, we conducted a 6 (blocks: 1–6) × 3 (study–test interval: 500 ms, 2,000 ms, 5,000 ms) ANOVA on accuracy data. The block factor was treated as a within-subjects variable, while the study–test intervals factor was a between-subjects variable and was balanced across participants with 13 participants in each group, respectively for 500 ms, 2,000 ms, or 5,000 ms.

For the analysis of response times, a one-way ANOVA examined the effect of study–test interval (500 ms, 2,000 ms, 5,000 ms). This analysis included only correct trials with response times between 200 ms and 2,000 ms.

Significant findings were followed by Bonferroni-corrected post hoc comparisons. A significance level of *p* <.05 was adopted for all statistical tests. To avoid drawing conclusions based solely on null results that might be observed due to chance, we utilized Bayesian analyses (Rouder et al., 2009). Bayesian analyses were conducted using JASP (Version 0.7.1.12) with default settings, which rely on Bayes factor calculations based on fixed-effects models using the Bayesian information criterion (BIC) approximation. For repeated-measures ANOVAs, JASP computes Bayes factors (BF₁₀) by comparing a model that includes the effect of interest (e.g., block, study–test interval, or their interaction) against a model that omits that specific effect while retaining the other effects in the design. In the case of an interaction term, the comparison is made between a model that includes the interaction and its associated main effects and a model that includes only the main effects, omitting the interaction. For Bayesian *t* tests, BF₁₀ reflects the evidence in favor of the alternative hypothesis (i.e., a difference between conditions) relative to the null hypothesis (i.e., no difference). This approach enables evaluation of the strength of evidence for or against specific effects, beyond reliance on *p* values. All Bayes factors reported in the manuscript were obtained using this procedure. The BF_10_ was employed to compute an odds ratio for the alternative hypothesis compared to the null hypothesis (values < 1 favor the null hypothesis, while values > 1 favor the alternative hypothesis). This approach provides a more nuanced assessment of the evidence for or against the alternative hypotheses by considering both the data and prior information.

In addition, we divided participants into one group who reported that they had noticed the array repetition, and a second group who reported not noticing the repetition. We report here the analyses of group data. The plots of data for individual participants are presented in Supplementary Material, Fig. S3-4. Estimates of the trial block in which participants had become aware of the repetition and their confidence in these estimates were retrospective and subjective, and did not appear to coincide with when participants started to improve performance. So we were not confident as to their reliability, and these data were not considered further.

For the following analyses, the Greenhouse–Geisser correction was employed to deal with violations of sphericity when necessary and adjusted degrees of freedom are reported. Partial eta square (η_p_^2^) values obtained from the ANOVA are reported for effect sizes.

### Results

#### Accuracy

**Fig. 2:**
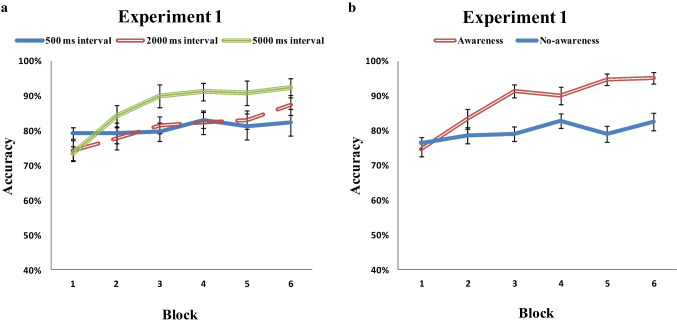
Accuracy comparing overall performance in Experiment 1 across blocks: (**a**) between 500 ms, 2,000 ms, and 5,000 ms memory–test interval tasks and (**b**) between awareness groups. Error bars represent standard errors of the mean. (Color figure online)

Means for accuracy across Blocks and Study–Test Intervals are illustrated in Fig. 2a, from which it is clear that the analysis was not confounded with ceiling or floor effects in the data. Accuracy analysis yielded a significant main effect of block, *F*(5, 180) = 11.94, *p* <.001, η_p_^2^ = 0.25, BF_10_ > 1,000, and a nonsignificant main effect of study–test interval, *F*(2, 36) = 2.57, *p* =.09, η_p_^2^ = 0.13, BF_10_ = 0.92. The Block × Interval interaction was significant, *F*(10, 180) = 2.32, *p* =.02, η_p_^2^ = 0.11, BF_10_ = 2.79. Pairwise comparisons revealed that the interaction was driven by the difference in performance across blocks for longer study–test intervals. More specifically, accuracy for the 5,000 ms study–test interval task improved significantly in Blocks 2 to 6 versus Block 1, that is after around 40 trials. Accuracy for the 2,000-ms study–test interval task improved significantly in Blocks 3 to 6 versus Block 1, that is after around 60 trials as in Shimi and Logie (2019), Experiment 1 in which the same study–test interval was used. In contrast, performance did not differ consistently across blocks in the 500-ms study–test interval task, except that Block 4 was higher compared with Block 1. Significant results of pairwise comparisons are shown in Supplementary Material, Table S3. As noted earlier, to allow direct comparison with previous relevant studies, our main analyses were conducted using 20-trial blocks. Given our interest in identifying when learning occurred across the different study–test interval conditions, we also performed a finer grained analysis using 10-trial blocks. This additional analysis, reported in the Supplementary Material, Fig. S5, produced a pattern of results consistent with the 20-trial block analysis.

We also analyzed the data separately for color change trials, shape change trials, and no-change trials; full details are reported in the Supplementary Materials,Fig. S6 and Table S4-5. Briefly, performance improved over trials for both change types but not for no-change trials, and these patterns were consistent across study–test intervals.

These results are consistent with the prediction that longer study–test intervals allow for greater consolidation of an episodic memory trace than shorter intervals, although even with a 5,000-ms interval, performance improvements occur only after 20 repetition trials. This is consistent with the proposal that a weak episodic memory trace is formed on each repetition and that around 30 repetitions of the same array with 5,000-ms consolidation time are required before the episodic trace becomes strong enough to start supporting performance. A 500-ms study–test interval appears to be too short to allow sufficient accumulation of an episodic representation even after 120 repetition trials.

Results also are consistent with our hypothesis of performance being supported by a limited capacity visual cache trial to trial, throughout all trials with a 500-ms study–test interval, and across early trials with longer study–test intervals until a strengthening episodic representation is sufficient to support performance and subsequent learning. However, it is unclear from the analyses so far whether the results are being driven by a bias in the type of response that generates false alarms or misses. To investigate this possibility, we carried out an analysis of sensitivity (*d*-prime) and bias (beta).

#### Signal-detection analyses

**Fig. 3:**
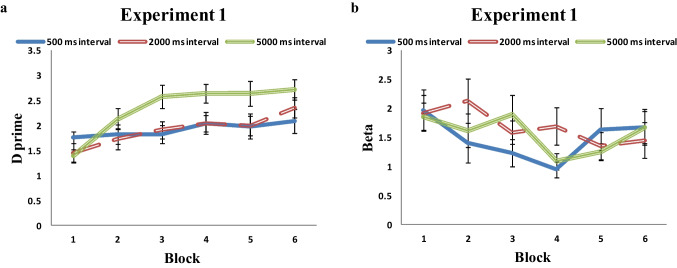
Values for *d*-prime (**a**) and beta (**b**) for study–test intervals of 500 ms, 2,000 ms, and 5,000 ms across trial blocks in Experiment 1. *Note*. In this change-detection task, β > 1 indicates a bias toward responding “no change,” whereas β < 1 reflects a bias toward responding “change.” This convention is the reverse of that typically used in old/new recognition paradigms (see Stanislaw & Todorov, 1999). (Color figure online)

To investigate whether results were due to sensitivity to detect a change or evidence of bias to respond “change” or “no change” we conducted a 6 (blocks: 1–6) × 3 (study–test interval: 500 ms, 2,000 ms, 5,000 ms) repeated-measures ANOVA for *d-*prime and beta. The *d*-prime analysis (Fig. 3a) yielded a significant main effect of block, *F*(5, 180) = 13.06, *p* <.001, η_p_^2^ = 0.27, BF_10_ > 1,000, but no significant main effect of study–test interval, *F*(2, 36) = 2.83, *p* =.07, η_p_^2^ = 0.13, BF_10_ = 1.08. The Block × Interval interaction was significant, *F*(10, 180) = 2.25, *p* =.03, η_p_^2^ = 0.11, BF_10_ = 2.25. This results pattern was the same as for percentage accuracy, and is consistent with the finding that participants gradually became more sensitive to the presence of a change on change trials, with increase in sensitivity largest for the 5,000-ms study–test interval.

The analysis for beta (Fig. 3b) revealed a significant main effect of block, *F*(5, 180) = 3.15, *p* =.01, η_p_^2^ = 0.08, BF_10_ = 2.27, but no significant main effect of study–test interval, *F*(2, 36) = 0.300, *p* =.74, η_p_^2^ = 0.02, BF_10_ = 0.17, nor a significant interaction between block and study–test interval, *F*(10, 180) = 1.53, *p* =.13, η_p_^2^ = 0.08, BF_10_ = 0.34. As is clear from Fig. 3b, there was a bias toward responding “no change” over the first two blocks of trials, after which this bias decreased, consistent with improvement in sensitivity to detect a change when it occurred. However, the “no change” bias remained in Block 6 after 120 trials.

The signal-detection analysis indicates that sensitivity (Fig. 3a) to detect a change mirrors the percentage accuracy data with no overall increase in *d*-prime for a 500-ms study–test interval, a slow increase for 2,000 ms, but a clear increase within the first two trial blocks for 5,000 ms followed by no change thereafter. Figure 3b shows that there was an overall bias to respond “no change” across trials, although this showed a modest reduction towards less bias over trials. This bias likely contributed to the relatively high performance on no-change trials across all blocks; however, high sensitivity (*d*-prime) also suggests that participants were generally able to distinguish changes from repetitions with reasonable accuracy. That is participants were unable to detect a change over the first block of trials, and this generated a no-change bias in responding even if some or all participants showed no evidence that they were aware of the repetition across the first two or three blocks. Initially, this bias would have led to error responses (misses) on change trials, but as sensitivity increased to detect a change, these “miss” responses gradually reduced. However, the lack of a Block × Study–Test Interval interaction for beta suggests that the advantage shown for a 5,000-ms interval cannot be the result of changes in response bias.

#### Self-reported awareness of repetition

Additional analyses were conducted based on participants’ self-reported awareness of the repetition in the memory array. Participants were categorized into two groups comprising those reporting awareness of the array repetition and those who reported no awareness of the repetition. For the 500-ms study–test interval, only one of the 12 participants reported awareness of the repetition. For the 2,000-ms study–test interval, five of the 13 participants reported awareness of the repetition. In the 5,000-ms study–test interval task, nine of the 13 participants reported awareness of the repetition.

We conducted a mixed 6 (blocks: 1 to 6) × 3 (study–test intervals: 500 ms vs. 2,000 ms vs. 5,000 ms) × 2 (awareness: awareness vs. no awareness) repeated-measures ANOVA on accuracy, with block as a within-subjects factor and study–test interval and reported awareness as between-subjects factors. The analysis revealed a significant main effect of block, *F*(5, 165) = 7.50, *p* <.001, η_p_^2^ = 0.18, BF_10_ > 1,000, main effect of reported awareness, *F*(1, 33) = 5.55, *p* =.03, η_p_^2^ = 0.14, BF_10_ = 32.14, and a significant interaction between block and reported awareness, *F*(5, 165) = 3.28, *p* =.01, η_p_^2^ = 0.90, BF_10_ = 66.60, but no significant main effect of study–test interval, *F*(2,33) = 0.55, *p* =.58, η_p_^2^ = 0.03, BF_10_ = 0.97, no significant interaction between reported awareness and study–test interval, *F*(2, 33) = 0.41,* p* =.67, η_p_^2^ = 0.02, BF_10_ = 0.39, no significant interaction between block and study–test interval, *F*(10, 165) = 0.95, *p* =.49, η_p_^2^ = 0.05, BF_10_ = 0.14, and no significant interaction between block, study–test interval and reported awareness, *F*(10, 165) = 0.99, *p* =.46, η_p_^2^ = 0.06, BF_10_ = 0.20.

Because no significant main effect of study–test interval or significant interactions involving the study–test interval, were found, and the numbers in each cell of the design were small and uneven, we did not analyze results separately for different study–test intervals. Results of pairwise comparisons on significant results are illustrated in Fig. 2b, and detailed in Supplementary Material, Table S6. These showed that performance for the “awareness” group improved significantly in Blocks 2 to 6 compared with Block 1. In contrast, there were no statistically significant differences across Blocks in the “no-awareness” group except between Blocks 1 and 4, and between Blocks 1 and 6.

#### Response time

**Fig. 4:**
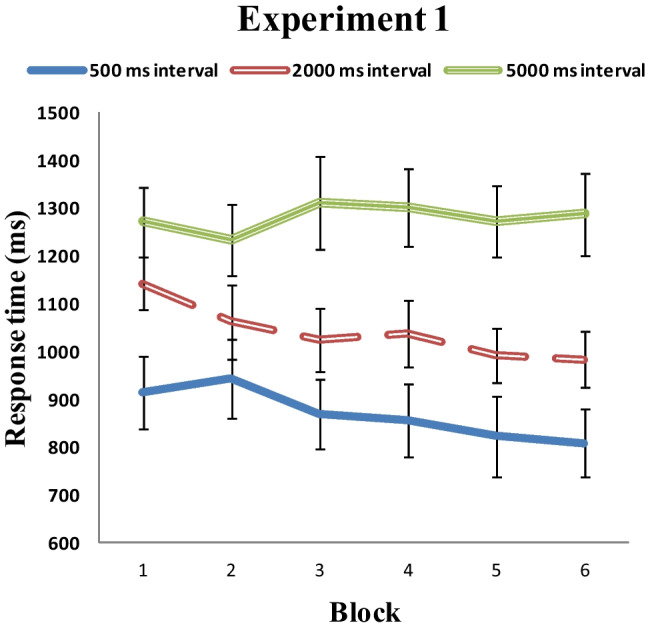
Mean correct response times across trial blocks and study–test intervals in Experiment 1. (Color figure online)

Participants were instructed to focus on responding accurately, with no emphasis on speed of responding. However, we analyzed the response time data, primarily to check if there was any evidence of speed–accuracy trade-offs, and to investigate whether the variation in study–test intervals, and consequent variation in intertrial intervals had an impact on correct response times. Figure 4 illustrates these data.

The analysis for response time yielded significant main effects of block, *F*(5, 180) = 2.76, *p* =.02, η_p_^2^ = 0.07, BF_10_ = 1.00, and of study–test interval, *F*(2, 36) = 9.01, *p* =.001, η_p_^2^ = 0.33, BF_10_ = 38.99. The Block × Interval interaction was not significant, *F*(10, 180) = 1.77, *p* =.10, η_p_^2^ = 0.09, BF_10_ = 0.57. These results indicate that response times were slowest for the longest study–test intervals, and fastest for the shortest study–test interval. Response times also became faster over time, but the lack of an interaction suggests that the accuracy advantage for 5,000 ms that developed over trials was not because responses were becoming slower. That is, there was no speed–accuracy trade-off.

### Discussion

The results of Experiment 1 showed clearly that with a study–test interval of 5,000 ms the overall group data showed improvements in performance after around 40 repetitions of the memory array. This was driven by the data from nine of the 13 participants in this group who reported awareness of the repetition. With a study–test interval of 2,000 ms, performance improvements in the group data began after 60 repetitions, with five of the 13 participants reporting awareness of the repetition: This replicated the data pattern found with the same study–test interval in Shimi and Logie (2019). With a study–test interval of 500 ms there was no evidence of performance improvement across repetitions except for an increase after 80 trials that did not increase further after 100 or 120 trials. Only one of the 13 participants in this group reported becoming aware of the repetition.

Although participants were told to focus on accuracy, the analysis of response times showed that there was no evidence that participants were improving accuracy at the expense of speed of responding, and overall response times became faster across trials. When given a longer study–test interval, participants tended to have slower response times. A possible account is that because the longer interval allowed for a greater focus on attempting to encode the array in episodic memory, and presentation of the test array disrupted that process, this led to a longer time to decide if the test array matched the study array. With a shorter study–test interval there was very little time for episodic encoding before the test array appeared, so there was no encoding process to disrupt. However, this is somewhat speculative, and we will return to possible alternative accounts in the General Discussion.

These results are entirely consistent with the hypothesis that increasing the study–test interval for change detection with a repeated visual array allows for greater consolidation of the repeated array in episodic memory, and generates performance improvements, but only after 40 or more repetitions and only with longer study–test intervals. Results suggest that performance improvements arose from increasing sensitivity to detect a change, coupled with reduction in a bias to respond “no change.” These performance improvements did not occur with a study–test interval of 500 ms, but from the first trial block and throughout all the trials participants in this group appeared to be able to store the color, shape, and location combinations for, on average, four of the six items. This finding is consistent with the hypothesis that a limited capacity visual cache can store around four items, and this is independent of long-term episodic memory. However, a study–test interval of 500 ms appears to be insufficient time to consolidate or strengthen an episodic trace for the full six-item array before the next trial overwrites the contents of the cache, even if the following stimulus array is identical. As a result, there is no evidence of learning of an array that is presented across 120 repetition trials when tested with recognition. With 5,000-ms or 2,000-ms study–test intervals, again performance on the first trial block indicates that four items can be retained before any learning has occurred, then performance starts to improve respectively after 40 or 60 repetition trials as there is gradual learning of the repeated array. However, this improvement occurs only for those participants for which the episodic trace strengthens sufficiently to support learning. The longer the interval, the greater the consolidation on each trial, and the faster the strengthening episodic trace begins to support change detection (recognition) performance to complement the use of the limited capacity visual cache. Longer intervals also make postexperimental reports of awareness of the repetition more likely.

## Experiment 2

In previous studies there was no improvement in performance when a different stimulus was presented on each trial. This is true whether memory was tested by recall or by recognition (e.g., Araya et al., 2024; Hebb, 1961; R. H. Logie et al., 2009; Olson & Jiang, 2004; Page et al., 2013; Souza & Oberauer, 2022). However, those earlier studies tended to have limited numbers of trials with the novel items, and often these were presented between trials with a repeated stimulus. If it is the case that the repeated array in Experiment 1 was overwritten by the array on the next trial, then we should find that presenting a novel array on every trial should generate data that look very similar to the results found for participants who were unaware that the same array was repeated, particularly with a short (500 ms) study–test interval. Alternatively, at least some of the performance improvements observed in Experiment 1 could have come from some of the participants simply getting better with change detection from practice on the task across 120 trials. Previous studies with novel arrays might have had too few trials for any practice effects to emerge. If this was the case, then we should observe performance improvements across trial blocks even when the memory array is different on every trial. These possibilities were tested in Experiment 2.

### Method

#### Participants

In Experiment 2, a new group of 39 college students (29 women, 10 men; *M* = 19.82, *SD* = 1.34) participated for a small honorarium. This sample size is sufficient to detect a moderate-to-small effect size (e.g., η_p_^2^ = 0.10) with 80% statistical power at a significance level of 0.05 based on a power analysis (Faul et al., 2007) with a 6 (blocks: 1 to 6) × 3 (study–test intervals: 500 ms vs. 2,000 ms vs. 5,000 ms) design for a repeated-measures ANOVA. All participants reported having normal or corrected-to-normal vision and normal color vision and provided written informed consent prior to participating in the study. Ethical approval for the study was obtained from the ethics committee of Sichuan Normal University.

#### Apparatus and stimuli

The experimental design of Experiment 2 mirrored that of Experiment 1, adhering to a 6 (blocks: 1 to 6) × 3 (study–test intervals: 500 ms vs. 2,000 ms vs. 5,000 ms) repeated-measures factorial design. The block factor was treated as a within-subjects variable, while the study–test intervals factor was a between-subjects variable and was balanced across participants with 13 participants in each group, respectively for 500 ms, 2,000 ms, or 5,000 ms. The only difference between experiments was that, in Experiment 2, the six-item array involved different combinations of colors, shapes, and locations on every trial.

#### Procedure

The procedure for Experiment 2 was identical to that of Experiment 1.

#### Statistical design and analyses

The statistical design and analyses for Experiment 2 was the same as Experiment 1.

### Results

#### Accuracy

**Fig. 5:**
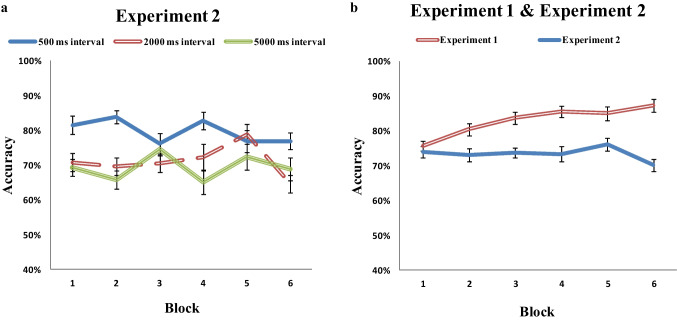
Mean percentage accuracy across trial blocks, and study test intervals of 500 ms, 2,000 ms, and 5,000 ms in (**a**) Experiment 2 and in (**b**) comparing Experiment 1 and Experiment 2 collapsing over study–test intervals. Error bars represent standard errors of the mean. (Color figure online)

Mean accuracy levels across blocks for study–test intervals 500 ms vs. 2000 ms vs. 5000 ms are illustrated in Fig. 5a. The analysis for accuracy yielded a significant main effect of study–test intervals, *F*(2, 36) = 14.59, *p* <.001, η_p_^2^ = 0.45, BF_10_ = 318.66, and a significant interaction between block and study–test intervals, *F*(10, 180) = 2.56, *p* =.01, η_p_^2^ = 0.13, BF_10_ = 8.09, but no significant main effect of block, *F*(5, 180) = 1.55, *p* =.18, η_p_^2^ = 0.04, BF_10_ = 0.11.

Participants in the 500-ms condition (*M* = 0.80, *SD* = 0.04) showed significantly better performance than those in the 2,000-ms condition (*M* = 0.71, *SD* = 0.07), *t*(24) = 3.96, *p* <.001, Cohen’s *d* = 1.55, BF_10_ = 47.55 and also showed significantly better performance than those in the 5,000-ms condition (*M* = 0.69, *SD* = 0.05), *t*(24) = 6.59, *p* < 0.001, Cohen’s *d* = 2.59, BF_10_ > 1000. No significant difference in overall memory performance was found between the participants in the 2,000-ms and 5,000-ms conditions, *t*(24) = 0.80, *p* =.43, Cohen’s *d* = 0.31, BF_10_ = 0.46. To further explore the significant Block × Study–Test Interval interaction observed in the accuracy data, we conducted simple comparisons between Block 1 and each of the subsequent blocks (Blocks 2–6) separately for each interval condition. These results revealed that, despite the significant interaction, only two comparisons reached statistical significance: performance in the 2,000-ms condition was significantly lower in Block 6 (*M* = 0.65, *SD* = 0.09) compared with Block 1 (*M* = 0.71, *SD* = 0.09), *t*(12) = 2.26, *p* =.043, Cohen’s* d* = 0.63, BF₁₀ = 1.80; performance in the 5,000-ms condition was significantly higher in Block 3 (*M* = 0.75, *SD* = 0.07) compared with Block 1(*M* = 0.69, *SD* = 0.09). Other comparisons yielded nonsignificant results, and most Bayes factors provided anecdotal or moderate evidence supporting the null hypothesis (BF₁₀ < 1). These findings suggest that the observed interaction was not driven by consistent improvements over time, and they reinforce our interpretation that no clear learning effect emerged in Experiment 2, even with extended study–test intervals. Full details of these analyses are presented in Supplementary Material, Table S7.

We also divided the data according to the three trial types: color change trials, shape change trials, and no-change trials. However, because the main effect of block was not significant, we report this analysis in Supplementary Material, Fig. S7.

#### Signal-detection analysis

**Fig. 6:**
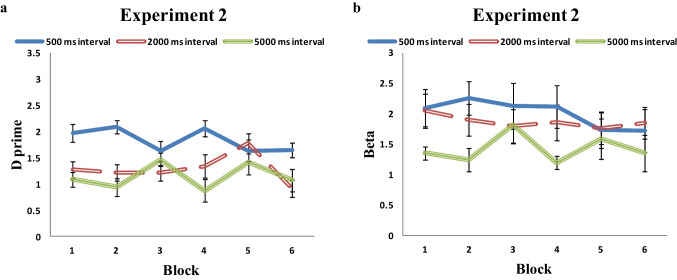
Values for *d*-prime (**a**) and beta (**b**) for study–test intervals of 500 ms, 2,000 ms, and 5,000 ms across trial blocks in Experiment 2. (Color figure online)

We conducted a 6 (block: 1–6) × 3 (study–test interval: 500 ms, 2,000 ms, 5,000 ms) repeated-measures ANOVA for *d*-prime. This yielded a significant main effect of study–test interval, *F*(2, 36) = 15.27, *p* <.001, η_p_^2^ = 0.46, BF_10_ = 525.90, and a significant interaction between block and study–test interval, *F*(10, 180) = 2.60, *p* =.01, η_p_^2^ = 0.13, BF_10_ = 8.65, but no significant main effect of block, *F*(5, 180) = 1.79, *p* = 0.12, η_p_^2^= 0.05, BF_10_ = 0.16. This pattern of results, illustrated in Fig. 6a, was the same as that for accuracy.

We also conducted a 6 (block: 1–6) × 3 (study–test interval: 500 ms, 2,000 ms, 5,000 ms) repeated-measures ANOVA for Beta. The analysis revealed no significant main effect of block, *F*(5, 180) = 0.65, *p* =.66, η_p_^2^ = 0.02, BF_10_ = 0.03, and no significant main effect of study–test interval, *F*(2, 36) = 2.27, *p* =.12, η_p_^2^ = 0.11, BF_10_ = 0.81. The interaction between block and study–test interval was also nonsignificant, *F*(10, 180) = 0.93, *p* =.50, η_p_^2^ = 0.05, BF_10_ = 0.08. Results are illustrated in Fig. 6b.

The *d*-prime analysis indicates that there was greater sensitivity to detect a change with a short (500 ms) study–test interval than with longer intervals, and although there was some variation there is no consistent change across trial blocks. The analysis of Beta suggests that there was a bias to respond “no change” as in Experiment 1, but unlike Experiment 1, there is no systematic reduction in this bias across trials.

#### Comparison of experiments

An additional 6 (blocks: 1 to 6) × 3 (study–test interval: 500 ms, 2,000 ms, and 5,000 ms) × 2 (experiment: 1 vs. 2) mixed-model repeated-measures ANOVA was conducted on accuracy, considering block as a within-subject factor and study–test interval and experiment as between-subject factors. The results, illustrated in Fig. 5b, revealed a significant main effect of block, *F*(5, 360) = 4.48, *p* <.001, η_p_^2^ = 0.06, BF_10_ = 6.36, main effect of experiment, *F*(1, 72) = 39.69, *p* <.001, η_p_^2^ = 0.34, BF_10_ > 1,000, significant interaction between block and study–test interval, *F*(10, 360) = 3.01, *p* <.001, η_p_^2^ = 0.08, BF_10_ = 14.30, significant interaction between block and experiment, *F*(5, 360) = 6.83, *p* <.001, η_p_^2^ = 0.09, BF_10_ > 1,000, and a significant interaction between experiment and study–test interval, *F*(2, 72) = 9.96, *p* <.001, η_p_^2^ = 0.22, BF_10_ = 158.12, and no significant interaction between block, study–test interval and experiment, *F*(10, 360) = 1.92, *p* =.05, η_p_^2^ = 0.05, BF_10_ = 0.99, but no significant main effect of study–test interval, *F*(2,72) = 2.51, *p* =.09, η_p_^2^ = 0.07, BF_10_ = 0.31. Additionally, a comparison of memory performance across each block in both experiments indicated no significant difference in Block 1. However, participants in Experiment 1 exhibited significantly better performance than those in Experiment 2 in Block 2, Block 3, Block 4, Block 5, and Block 6. Results of pairwise comparisons on significant results are given in Supplementary Material, Table S8.

As for Experiment 1, upon completion of the experimental trials, participants were asked whether they perceived the stimuli presented in the memory array as the same on each trial or as different on each trial. All participants indicated that they perceived the memory array as distinct on every trial.

#### Response time

**Fig. 7:**
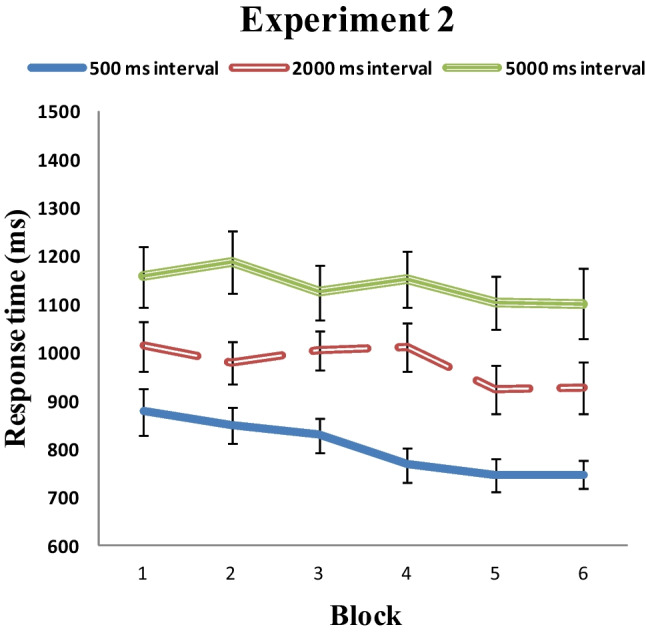
Mean correct response times across trial blocks and study–test intervals of 500 ms, 2,000 ms, and 5,000 ms in Experiment 2. (Color figure online)

Finally, we analyzed response time data for each of the three study–test intervals across blocks, illustrated in Fig. 7.

The response-time analysis yielded significant main effects of block, *F*(5, 180) = 5.20, *p* <.001, η_p_^2^ = 0.13, BF_10_ = 125.85, and of study–test intervals, *F*(2, 36) = 15.67, *p* <.001, η_p_^2^ = 0.47, BF_10_ = 1455.72. The Block × Intervals interaction was not significant, *F*(10, 180) = 0.80, *p* =.59, η_p_^2^ = 0.04, BF_10_ = 0.05. These results indicate that response times were slowest for the longest study–test intervals, and fastest for the shortest study–test interval, consistent with Experiment 1, suggesting that the effects of study–test interval on response time did not arise because the stimulus array was repeated across trials. Response times also became faster over trials, as in Experiment 1. This also suggests a practice effect across trials in time to respond (but not in accuracy) that does not depend on repeating the stimulus arrays. The key difference between experiments was the slow change in accuracy across 120 trials for 5,000-ms and 2,000-ms study–test intervals in Experiment 1 but not in Experiment 2. Neither experiment resulted in performance improvements with a 500-ms study–test interval.

### Discussion

In Experiment 2 there was no evidence of consistent improvement in performance across blocks of trials for any of the three study–test intervals included. This rules out a general effect of practice on accuracy across trials with our change detection test in Experiment 1. From Fig. 5a, it is clear that the lack of performance improvement in this experiment was very similar to that illustrated in Fig. 2a (500-ms group) and Fig. 2b (not aware) for participants in Experiment 1 who showed no improvement across trials. That is, participants showing no improvement in Experiment 1 were responding on each trial as if the study array was different on every trial, indicating that they had no episodic trace from the previous trial to support learning from the repetition or for them to become aware of the repetition.

Performance for 2,000-ms and 5,000-ms study–test intervals was at around 70% from trial Block 1 and showed little or no systematic increase between Block 1 and Block 6, indicating that participants continued to rely on a limited capacity memory system across all trials. There was some unexpected variability between Blocks 1 and 3 for 5,000 ms and between Block 1 and Block 6 for 2,000 ms, but neither of these findings offer evidence for consistent learning across trials. There was also no increase in performance across trials with a 500-ms study–test interval. However, overall performance was better for 500 ms than for 2,000 ms or 5,000 ms with no overall difference between the latter two. It is possible that the longer intervals allowed for some forgetting of the temporary memory for the array, or because participants were encouraged to focus on accuracy, then the long-study–test intervals led to less urgency in the need to respond. However, there is no evidence of a speed–accuracy trade-off. That is, participants did not take longer to respond in order to improve their accuracy. Instead, longer response times occurred with the longer study–test intervals for which accuracy was poorer.

Notable too was the finding of a “no change” response bias, suggesting that this same bias in Experiment 1 was unlikely to be due to the repetition of the stimulus array. Likewise, improvements in response time across trials occurred in both experiments.

## General discussion

Our aim was to explore further a previous finding from Shimi and Logie (2019; R. H. Logie et al., 2009; Olson & Jiang, 2004) that up to 120 repeated presentations of exactly the same visual array of six items, with participants not being informed about the repetition, resulted in no, or very slow performance improvements when memory was tested with change detection or other tests of recognition. This was in striking contrast with a wide range of studies that have shown learning of repeated presentation and recall of sequences of verbal and visual material with, in some cases, fewer than 10 repetitions of the target stimulus (e.g., Couture & Tremblay, 2006; Hebb, 1961; Hitch et al., 2005; Page et al., 2006, 2013; Sukegawa et al., 2019; Turcotte et al., 2005).

A related previous finding in Shimi and Logie (2019) was that repeated presentation of an array of four items resulted in near ceiling performance during the first few repetitions and throughout the remaining trials. Shimi and Logie (2019) suggested that a visual cache memory (R. H. Logie, 1995) could support temporary retention of around four items within a single trial, but that the contents of the visual cache were overwritten by the study array on the next trial, even if the array was identical on every trial. With six-item arrays, this resulted in performance at around 70%, indicating accurate retention in the visual cache of just four of the six items, again with the contents of the cache overwritten by the array on the next trial, even although the arrays were identical. There was evidence that after around 60 repetitions of the same six-item array, some participants started to show performance improvements, some other participants showed improved performance after more than 100 repetition trials, and some showed no improvements after 120 repetition trials. None of the groups reached ceiling performance even after 120 repetition trials.

Following a previous proposal by R. H. Logie (1995, 2003, 2011), Shimi and Logie (2019) suggested that, in addition to the temporary, limited capacity visual cache supporting retention of the stimulus only for the duration of each single trial, a very weak trace of the array was formed in episodic long-term memory, and this trace very slowly strengthened with each repeated presentation. However, 60 repetition trials were required before the episodic memory trace was sufficiently strong to allow performance to be supported on each trial by both four items from the visual cache and perhaps five or six items (including those also in the visual cache) from episodic memory. With further trials, it was assumed that performance was increasingly supported by episodic memory, and gradually less supported by the visual cache, providing evidence for slow learning of the repeated array across 120 trials. This interpretation was also consistent with the view that for any given working memory task, there may be more than one cognitive function that contributes to the performance observed (e.g., Adam et al., 2024; Baddeley, 1986; Baddeley & Hitch, 1974; R. H. Logie, 1995, 2003, 2011, 2018, 2023; Oberauer, 2009; Oberauer et al., 2017).

Experiment 1 investigated the possibility that the 2000 ms study–test interval in Shimi and Logie (2019) might have been insufficient to allow for consolidation of the array in episodic memory within the time course of a trial, and hence only a weak episodic trace was formed or marginally strengthened on each trial. This hypothesis was supported by the finding that, with a 5,000-ms study–test interval, performance improved in the group data across the first 40 trials. In contrast, with a 500-ms study–test interval there was no evidence of any performance improvement across 120 trials. Performance was at around 80% across all trials, indicating the retention on every trial of only four or five of the six items despite the large number of repeated presentations. With a 2,000-ms study–test interval, the pattern was very similar to the data in Shimi and Logie (2019), with performance improvements in the group data after around 60 trials, and continued slow improvements thereafter but a failure to reach ceiling, even after 120 trials. It is not clear if any learning that was observed resulted only from repeated presentation of the study array, or also from repeated presentation during half of the test arrays (no-change trials). If both contributed, then it could be argued that over 90 repetitions (60 study arrays and 30 test arrays) were required before performance started to improve for participants who reported becoming aware of the repetitions in Shimi and Logie (2019), and in Experiment 1 for the 2,000-ms study–test interval, and 60 repeated presentations for the 5,000-ms interval. On this argument, even after seeing the same stimulus array 180 times (120 study arrays and 60 test arrays) all but one of the participants in the 500 ms group showed no evidence of learning, and reported not being aware of the repetition.

Experiment 2 found no improvements in performance across trials with a novel array on every trial, and for all three study–test intervals. Moreover, for all three conditions performance on the first block of trials was approximately the same in Experiments 1 and 2. With regard to our primary motivation for Experiment 2, the level and pattern of performance for the 500-ms study–test interval was very similar in both experiments. This suggests that participants in this condition in Experiment 1 were responding as if the stimulus was different on every trial. The overall performance level of 70%–80% is consistent with our interpretation that performance was based on retention of four or five items from the array being held in a temporary visual cache memory to support performance within each trial, but with insufficient time in Experiment 1 to consolidate even a weak trace of the array into episodic long-term memory. There was therefore no residual trace between one trial and the next to support learning of the whole array.

With regard to our secondary motivation for Experiment 2, the results confirmed that the slow improvements in performance found in Experiment 1 could not be attributed to overall practice with the task across 120 trials.

Notable from Experiment 2 (Fig. 5), the baseline performance levels for both of the longer study–test intervals were lower than the baseline performance for the shortest (500 ms) interval, with no subsequent consistent evidence of learning. This was also true, for the initial baseline levels for Experiment 1 (Fig. 2). These observations suggest that there was greater loss, possibly due to decay, over delays of 2,000 ms and 5,000 ms between presentation of the study array and presentation of the test array, than there was for a 500-ms study–test delay. During the early trials of Experiment 1, no learning could have occurred, yet performance was well above chance for all three conditions. This suggests that performance on the early trials in Experiment 1, and through all trials in Experiment 2 was supported by a temporary memory system that did not depend on learning of the stimulus array. This is entirely consistent with our proposal of a temporary, limited capacity visual cache.

The finding in Experiment 1 that performance with the longer study–test intervals subsequently exceeded that for the 500-ms interval, but only after 40 or more repetitions of the study array, is consistent with our hypothesis of weak activation of an episodic trace on each trial that only occurs with a longer study–test interval, and only starts to support performance after accumulating strength from multiple repetitions.

The analyses of response times in both experiments confirmed that the accuracy data could not be explained by a speed–accuracy trade-off. The speeding of response time across trial blocks in both experiments suggested that this was a general practice effect with a change-detection task, and that getting faster in responding with practice was not dependent on presenting the same array repeatedly across trials. Likewise, a conservative bias towards responding “no change” occurred in both experiments, suggesting that this too did not arise because the array was repeated in Experiment 1.

The data from these two experiments are consistent with our hypothesis of two distinct memory systems: a limited capacity, temporary visual cache and episodic long-term memory both supporting performance on the change-detection task. However, there remain questions about what might be the limitations on episodic encoding that result in evidence for such slow learning despite a large number of repetitions of the same stimulus array.

As noted in the introduction, the results from previous studies, and now from Experiment 1, showing a lack of learning or very slow learning from recognition tests with repeated presentation of the same array (R. H. Logie et al., 2009; Olson & Jiang, 2004; Shimi & Logie, 2019; Souza & Oberauer, 2022) is difficult to explain in the context of the theoretical framework known as embedded processes (e.g., Cowan, 1999, 2023; Cowan et al., 2021). This assumes that the current contents of working memory comprise currently activated traces from long-term memory, coupled with a limited focus of attention at any one time on a small amount of the activated traces. On this basis, repeated presentation of the same combination of stimulus features should result in repeated activation of the same feature combinations which are then repeatedly in the focus of attention for the full duration of a trial (up to 6,000 ms). This should result in strengthening of the activated traces and performance improvements across repetitions showing evidence of learning. The complete lack of performance improvement across as many as 60 repeated presentations of the same array, and after even more repetitions for some participants, and not at all with a 500-ms study–test interval does not seem compatible with this account.

The embedded processes account could account for these data if the repeated activation of the presented feature combinations on each trial is very weak and insufficient to support performance or learning until incremental strengthening of that activation reaches a sufficient level for awareness of the repetition and subsequent learning. However, an additional assumption is also needed to account for performance levels above chance across multiple trials during which no improvements in performance are observed. This can be accommodated by assuming an additional assumption of a “peripheral” component of working memory that can store a small amount of visual information in a passive form on a temporary basis, and that is distinct from the focus of attention (e.g., Baddeley, 1986; Barrouillet & Camos, 2021). We propose that such a peripheral component can be considered a temporary visual cache, originally described as a limited capacity temporary visual short-term memory that can retain a small amount of visual information (such as a small single visual array) in a passive form for the few seconds of each trial, but that requires attention to refresh or rehearse its contents for retention over longer periods (R. H. Logie, 1995, 2003, 2011; R. H. Logie, Belletier, et al., 2021; see also Phillips & Christie, 1977). If not actively refreshed or rehearsed its contents are replaced a few seconds later by the stimulus on the next trial. The evidence from Olson and Jiang (2004), R. H. Logie et al. (2009), Shimi and Logie (2019), Souza and Oberauer (2022), and the current Experiment 1 suggest that the contents of the cache are replaced by the stimulus on the next trial even if that stimulus is identical. This suggests that with minor modifications of each theoretical framework, differences between embedded process and our account can be resolved and possibly account for a wide range of other apparently incompatible results (see discussions in Cowan et al., 2020; R. H. Logie, 2023; R. H. Logie, Belletier, et al., 2021) as well as the results from the current manuscript.

Another possible account suggested by one of the reviewers of an earlier version of this manuscript was to describe the effect of activation of an episodic trace as the basis for a priming effect that could boost performance on subsequent trials, with a focus on the intertrial intervals. If there was any form of short-term priming between one trial and the next, we might expect to see evidence of priming for short, but not for longer intertrial intervals. In order to equate overall time for each trial (6,000 ms), the intertrial interval varied inversely with the study–test interval. Therefore, the 500-ms study–test interval was followed by a 5,500-ms intertrial interval, and the 5,000-ms study–test interval was followed by a 1,000-ms intertrial interval. However, in neither case was there evidence of a short-term priming effect in which presentation of the stimulus on one trial boosted performance on the following trial. Only after many multiple repetitions was there any evidence of performance improvement.

However, the account proposed by Shimi and Logie (2019) in terms of a gradually strengthening, weak episodic trace might be interpreted as a form of accumulative priming across multiple trials. On this account the temporary visual cache supports retention of the stimulus array between study and test for the early blocks of trials, but the intertrial interval of 5,500 ms for the 500-ms study–test interval might have weakened the episodic activation before the following trial. Therefore, the activation was too weak to “prime” or improve performance with the same stimulus on the next trial, or was reduced to baseline activation before the next trial so that there was effectively no residual activation on which the activation on the next trial could build. Hence, there was no learning in this condition. There would be less weakening of this trace between trials with the shorter 1,000-ms intertrial interval for the 5,000-ms study–test interval, and so there would be more chance that, after 40 repetitions, the trace accumulated to a sufficient level to boost (prime) performance with incremental strengthening on subsequent trials to further enhance performance. This account is entirely compatible with our own suggestion of a weak episodic trace that results in learning by incremental strengthening across repetition trials, but that with a short study–test interval and a long intertrial interval the episodic trace does not form or is too weak to accumulate across trials. It may be that both the study–test interval and the intertrial interval place important constraints on the accumulation of episodic activation, but this account does not undermine our overall conclusions from the current set of data, and the relative contributions of study–test interval and intertrial interval could be explored in future studies.

A further possibility is that the capacity of a visual temporary (or short-term) memory, that we refer to as a visual cache, places constraints, or represents a “bandwidth” on what can be encoded long term. Fukuda and Vogel (2019) explored this hypothesis in some depth and concluded that the capacity of visual short-term memory does not predict whether or not visual information accumulates in long-term memory. They also found that time to encode does not seem crucial. This latter result contrasts with our own finding that a longer study–test delay resulted in better performance and learning after 40 repetitions when the stimulus was repeated across trials, but a shorter delay resulted in no performance improvements even with 120 repetitions of the same stimulus array. The Fukuda and Vogel result also contrasts with a growing literature on what is known as “event segmentation,” in which clear start and end points for an event, such as the boundaries of an experimental trial, can form the basis for encoding an episode as long as the amount of information is within the capacity of working memory (e.g., Güler et al., 2024; Kurby & Zacks, 2008; M. Logie & Donaldson, 2021, 2025). The contents of working memory are updated as a new event occurs, allowing us to keep track of rapid changes that we encounter throughout the day, while accumulating a trace of each event in episodic memory. Crucial within this literature is the role of “prediction errors,” in which a participant encounters something that they did not expect, and this triggers the episodic encoding of the event. The data presented here cannot address why these contrasting results appear, but they offer a possible route for future research on when episodic encoding does and does not occur and how this relates to the limited capacity of visual working memory.

We acknowledge that our a priori power analysis may have yielded an overly optimistic estimate for detecting a mixed (between–within) interaction with only 39 participants in total. As Brysbaert (2019) shows, split-plot interactions with effects in the typical range for psychology generally require on the order of ~100 to 200 participants in total to robustly detect the interaction and its supporting simple effects, and G*Power can return deceptively small sample sizes under assumptions that rarely hold in practice. More broadly, Brysbaert (2019) argues that, for many common designs, samples below ~100 per between-subjects group are typically underpowered, with mixed-design interactions often needing ~200 participants for 80% power. At the same time, statistical power is jointly determined by the number of participants and by the number of observations per participant per condition. In our study each participant contributed six repeated measurements (six blocks) per within-subject factor level, which increases the reliability of condition means and the correlation between repeated measures. This can boost the effective repeated-measures effect size (because higher within-person reliability raises *r* between levels). Indeed, Brysbaert (2019) suggests that moving from single to multiple observations per condition can markedly improve reliability and apparent effect sizes in repeated-measures designs, which helps stabilize parameter estimates even with modest *N*. Nevertheless, increasing observations per participant cannot fully substitute for larger samples when the critical variance component is between participants(as is often the case for mixed-design interactions), so residual risk of Type-M error and false positives remains higher in small-*N* mixed designs. Importantly, the central pattern we emphasize is not a fragile significant interaction but rather the absence of early-block learning across many repetitions, which conceptually replicates prior null-learning results using recognition/change detection (R. H. Logie et al., 2009; Olson & Jiang, 2004; Shimi & Logie, 2019; Souza & Oberauer, 2022). This cross-study convergence increases confidence that the early null interactions in our data reflect a genuine property of the underlying mechanisms rather than a mere artifact of insufficient power.

In conclusion, the results reported here are consistent with the proposal that performance on a visual recognition test can be supported on each trial by a limited capacity visual cache (R. H. Logie, 1995, 2003, 2011) the contents of which are overwritten by the visual stimulus on the next trial, even if that stimulus is identical. Evidence for this conclusion arises from the findings that, with a short (500 ms) delay between the study and test array for change detection, there is no evidence of learning of a six-item visual array even after it has been presented repeatedly for 120 trials (Experiment 1), and that the pattern of performance is the same as when the array is different on every trial (Experiment 2). With a longer study–test interval (5,000 ms) that allows for consolidation within each trial of a repeated visual array there is a gradual improvement in change detection performance, but only after the array has been repeatedly viewed over 40 trials. This is consistent with the gradual strengthening of a weak episodic trace in long-term memory, that begins to provide additional support to performance once it is sufficiently strong so to do. With a 500-ms study–test interval, any episodic trace is too weak to accumulate and provide support in addition to the visual cache. For some participants, even a 5,000-ms study–test interval fails to generate a sufficiently strong episodic record and so they rely on the visual cache throughout. The overall pattern of data supports the broader proposal (Hart et al., 2025; R. H. Logie, 1995, 2003, 2011, 2023; R. H. Logie et al., 2021) that there are potential contributions to working memory tasks from multiple cognitive functions and each participant will rely on the combination of those functions that can best maximise their performance on the task they are set.

## Supplementary Information

Below is the link to the electronic supplementary material.

Supplementary file1 (DOCX 321 KB)

## Data Availability

Anonymized data and materials are available on the Open Science Framework (OSF): https://osf.io/phbsu/

## Code availability

The code and programming scripts used in our study are publicly available on the Open Science Framework (OSF): https://osf.io/phbsu/
