# Supplementary material for: When repeated presentation of visual feature bindings does and does not result in learning: Visual short-term and long-term memory are distinct but work in tandem
Source: Mem Cognit. 2025 Nov 29;54(4):1388–406. doi: 10.3758/s13421-025-01816-8 (PMC13253647; doi:10.3758/s13421-025-01816-8)
Supplement: Supplementary file 1 — Supplementary file1 (DOCX 321 KB) [file 13421_2025_1816_MOESM1_ESM.docx]

**Supplementary Material**

**When repeated presentation of visual feature bindings does and does not result in learning: Visual short-term and long-term memory are distinct but work in tandem**

Chaoxiong Ye ^1,2^, Qiang Liu ^1,2,3*^ & Robert H Logie ^4^

^1^ School of Education, Anyang Normal University, China

^2^ Department of Psychology, University of Jyvaskyla, Finland

^3^ Institute of Brain and Psychological Sciences, Sichuan Normal University, China

^4^ Department of Psychology, University of Edinburgh, United Kingdom

*** Correspondence to:**

Qiang Liu, PhD

School of Education

Anyang Normal University, China

Email: lq780614@163.com

**Accuracy results with response time (RT) filtering**

To ensure the robustness of our findings, we conducted supplementary analyses by filtering trials based on response time (RT). Specifically, we excluded trials with RTs shorter than 200 ms or longer than 2000 ms, as such responses are commonly considered too fast to reflect deliberate processing or too slow to reflect focused attention. While the main text reports accuracy results based on all trials from the formal experiment, this supplementary analysis focused only on trials within the 200–2000 ms RT range.

The results obtained from this trimmed dataset were qualitatively identical to those from the full dataset. In particular, the main effects and interaction patterns in memory accuracy remained unchanged, supporting the reliability of the reported findings. Given that participants were instructed to prioritize accuracy over speed, and that the inclusion of extreme RTs did not distort the results, we chose to present the full dataset analyses in the main text for completeness.

The detailed results of the RT-filtered analysis are presented below.

**Experiment 1**

For Experiment 1, we report the analysis of accuracy, and mean accuracy across blocks for study–test intervals: 500 ms vs. 2000 ms vs. 5000 ms, illustrated in **Figure S1**. The analysis for accuracy yielded a significant main effect of block, *F*(5, 180) = 9.912, *p* < .001, *η_p_^2^* = .216, but a non significant main effect of study-test interval, *F*(2, 36) = 1.860, *p* = .170, *η_p_^2^* = .094. The Block × Intervals interaction was significant, *F*(10, 180) = 2.513, *p* = .018, *η_p_^2^* = .123. Pairwise comparisons revealed that the interaction was driven by the difference in performance across blocks for the longer study-test intervals. More specifically, accuracy for the longest 5,000 ms study–test interval task improved significantly in Blocks 2 to 6 compared with Block 1, that is after around 40 trials. Accuracy for the 2000 ms study–test interval task improved significantly in Blocks 3 to 6 compared with Block 1. In contrast, performance did not differ across blocks in the 500 ms memory–test interval task. Significant results of pairwise comparisons are shown in **Table S1**.

**Figure S1.** Accuracy with RT filtering comparing overall performance in Experiment 1 across blocks: (a) between 500 ms, 2000 ms and 5000 ms memory–test interval tasks. Error bars represent standard errors of the mean.

**Table S1.** Means (SDs) accuracy with RT filtering for each trial block, and pairwise block comparisons across study-test intervals (500 ms, 2,000 ms, 5,000 ms) in Experiment 1, along with t-value (*df* = 12, two-tailed p-value), *Cohen’s d*, and Bayes Factor (*BF₁₀*).

| **Interval** | **Block**  **Comparison** | **Block 1**  **Mean(SD)** | **Block X Mean(SD)** | **t** | **p** | **Cohen's d** | **BF10** |
| --- | --- | --- | --- | --- | --- | --- | --- |
| 500 ms | 1 vs. 2 | 0.804(0.022) | 0.802(0.031) | 0.056 | 0.956 | 0.016 | 0.279 |
| 500 ms | 1 vs. 3 | 0.804(0.022) | 0.815(0.030) | 0.436 | 0.671 | 0.121 | 0.302 |
| 500 ms | 1 vs. 4 | 0.804(0.022) | 0.824(0.029) | 0.861 | 0.406 | 0.239 | 0.382 |
| 500 ms | 1 vs. 5 | 0.804(0.022) | 0.812(0.041) | 0.239 | 0.815 | 0.066 | 0.285 |
| 500 ms | 1 vs. 6 | 0.804(0.022) | 0.829(0.036) | 0.837 | 0.419 | 0.232 | 0.375 |
| 2000 ms | 1 vs. 2 | 0.761(0.031) | 0.805(0.036) | 2.146 | 0.053 | 0.595 | 1.542 |
| 2000 ms | 1 vs. 3 | 0.761(0.031) | 0.828(0.028) | 2.832 | 0.015 | 0.786 | 4.145 |
| 2000 ms | 1 vs. 4 | 0.761(0.031) | 0.834(0.034) | 2.522 | 0.027 | 0.699 | 2.623 |
| 2000 ms | 1 vs. 5 | 0.761(0.031) | 0.845(0.027) | 2.826 | 0.015 | 0.784 | 4.107 |
| 2000 ms | 1 vs. 6 | 0.761(0.031) | 0.897(0.028) | 2.865 | 0.014 | 0.795 | 4.354 |
| 5000 ms | 1 vs. 2 | 0.730(0.019) | 0.843(0.019) | 2.256 | 0.044 | 0.626 | 1.796 |
| 5000 ms | 1 vs. 3 | 0.730(0.019) | 0.920(0.019) | 3.601 | 0.004 | 0.999 | 13.347 |
| 5000 ms | 1 vs. 4 | 0.730(0.019) | 0.912(0.018) | 3.675 | 0.003 | 1.019 | 14.926 |
| 5000 ms | 1 vs. 5 | 0.730(0.019) | 0.918(0.021) | 3.407 | 0.005 | 0.945 | 9.914 |
| 5000 ms | 1 vs. 6 | 0.730(0.019) | 0.934(0.019) | 4.250 | 0.001 | 1.179 | 35.772 |
|  |  |  |  |  |  |  |  |

**Experiment 2**

We report the analysis of accuracy in Experiment 2, and with mean accuracy across blocks for study–test intervals: 500 ms vs. 2000 ms vs. 5000 ms, illustrated in **Figure S2**. The analysis for accuracy yielded a significant main effect of study–test intervals, *F*(2, 36) = 9.699, *p* < .001, *η_p_^2^* = 0.350, and a significant interaction between block and study–test intervals, *F*(10, 180) = 2.128, *p* = .032, *η_p_^2^* = 0.106, but no significant main effect of block, F(5, 180) = 1.504, p = .191, *η_p_^2^*= 0.040. We compared overall memory performance across the three study–test intervals from Experiment 2 (Figure 2). Participants in the 500-ms condition (0.799 ± 0.041) showed significantly better performance than those in the 2,000-ms condition (0.722 ± 0.067), *t*(24) = 3.529, *p* = .002, and also showed significantly better performance than those in the 5,000-ms condition (0.715 ± 0.054), *t*(24) = 4.486, *p* < .001. No significant difference in overall memory performance was found between the participants in the 2000-ms and 5000-ms conditions, *t*(24) = 0.290, *p* = .775. Significant results of pairwise comparisons are shown in **Table S2**.

**Figure S2.** Accuracy with RT filtering comparing overall performance in Experiment 2 across blocks: (a) between 500 ms, 2000 ms and 5000 ms memory–test interval tasks. Error bars represent standard errors of the mean.

**Table S2.** Means (SDs) accuracy with RT filtering for each trial block, and pairwise block comparisons across study-test intervals (500 ms, 2000 ms, 5000 ms) in Experiment 2, along with t-value (*df*=12, two-tailed p-value), *Cohen’s d*, and Bayes Factor (*BF₁₀*).

| **Interval** | **Block**  **Comparison** | **Block 1**  **Mean(SD)** | **Block X Mean(SD)** | **t** | **p** | **Cohen's d** | **BF_10_** |
| --- | --- | --- | --- | --- | --- | --- | --- |
| 500 ms | 1 vs. 2 | 0.814(0.091) | 0.840(0.065) | 0.754 | 0.466 | 0.209 | 0.355 |
| 500 ms | 1 vs. 3 | 0.814(0.091) | 0.762(0.110) | 1.855 | 0.088 | 0.515 | 1.052 |
| 500 ms | 1 vs. 4 | 0.814(0.091) | 0.829(0.092) | 0.390 | 0.704 | 0.108 | 0.297 |
| 500 ms | 1 vs. 5 | 0.814(0.091) | 0.775(0.113) | 0.955 | 0.358 | 0.265 | 0.410 |
| 500 ms | 1 vs. 6 | 0.814(0.091) | 0.770(0.083) | 1.273 | 0.227 | 0.353 | 0.543 |
| 2000 ms | 1 vs. 2 | 0.715(0.100) | 0.709(0.097) | 0.264 | 0.796 | 0.073 | 0.287 |
| 2000 ms | 1 vs. 3 | 0.715(0.100) | 0.717(0.083) | 0.072 | 0.944 | 0.020 | 0.279 |
| 2000 ms | 1 vs. 4 | 0.715(0.100) | 0.731(0.122) | 0.406 | 0.692 | 0.112 | 0.299 |
| 2000 ms | 1 vs. 5 | 0.715(0.100) | 0.798(0.096) | 2.426 | 0.032 | 0.673 | 2.285 |
| 2000 ms | 1 vs. 6 | 0.715(0.100) | 0.658(0.102) | 1.962 | 0.073 | 0.544 | 1.207 |
| 5000 ms | 1 vs. 2 | 0.715(0.089) | 0.673(0.114) | 0.844 | 0.415 | 0.234 | 0.377 |
| 5000 ms | 1 vs. 3 | 0.715(0.089) | 0.765(0.076) | 1.939 | 0.076 | 0.538 | 1.172 |
| 5000 ms | 1 vs. 4 | 0.715(0.089) | 0.687(0.138) | 0.654 | 0.526 | 0.181 | 0.335 |
| 5000 ms | 1 vs. 5 | 0.715(0.089) | 0.737(0.147) | 0.460 | 0.654 | 0.128 | 0.305 |
| 5000 ms | 1 vs. 6 | 0.715(0.089) | 0.704(0.132) | 0.261 | 0.799 | 0.072 | 0.287 |

**Individual performance trajectories and reported awareness of repetition**

In Experiment 1, to further examine the relationship between participants’ subjective awareness of array repetition and their actual task performance, we plotted individual accuracy across the six experimental blocks. Participants were divided into two groups based on their response to a post-experiment question: those who reported noticing the repetition of the study array, and those who reported not noticing it.

These estimates were retrospective and subjective in nature. As shown in **Figure S3**, for the 500 ms study-test interval, the one participant who reported awareness improved across trials but was not consistent, and several participants appeared to improve while reporting no awareness while others showed no improvement or performance declined across trials. For 2000 ms there was no clear correspondence between performance improvement and reported awareness. For 5000 ms most, but not all participants reporting awareness improved performance across trials. However for those reporting no awareness, some showed improvement and some did not.

**Figure S3.** Individual accuracy across six blocks across study-test intervals (500 ms, 2000 ms, 5000 ms) in Experiment 1 for participants who reported noticing (blue) and not noticing (red) the repetition.


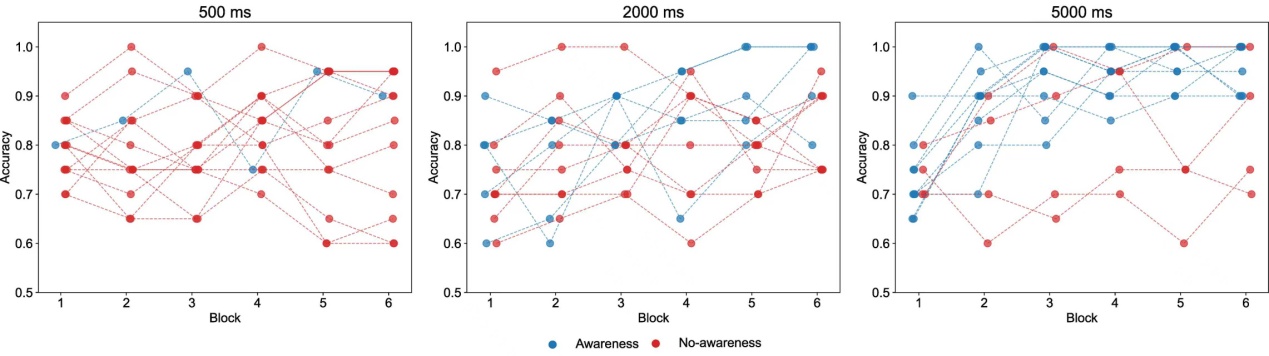


There was no relationship between the block of trials in which performance improvements were observed and the block estimated by participants as to when they had become aware of the repetition or the expressed confidence in this estimate.

Because of the lack of consistency between reported awareness of repetition and performance levels across trials we did not include the presentation and analysis of these data in the main text (the block-wise breakdown by interval and awareness is provided in **Figure S4**).

**Figure S4**. Accuracy performance in Experiment 1 as a function of study-test interval and self-reported awareness of repetition. we provide here the breakdown of accuracy performance across blocks by study-test interval separately for participants who reported awareness (a) and those who reported no awareness (b).

Due to the very small number of participants reporting awareness in the 500 ms condition (n = 1), a reliable statistical comparison between awareness groups across intervals and blocks was not possible. However, these visualizations help illustrate the performance patterns across conditions and support the interpretation that awareness of repetition may facilitate learning, particularly when the study-test interval is sufficiently long to allow episodic encoding.

**Pairwise Comparisons Across Blocks in Experiment 1**

To further explore the interaction between Block and Study–Test interval found in the accuracy analysis of Experiment 2, we conducted pairwise comparisons of accuracy across the six blocks within each Study–Test interval condition. This analysis was aimed at identifying whether performance changed systematically across blocks depending on the interval length.

The full results of all pairwise comparisons are presented in **Table S3**.

**Table S3.** Means (SDs) of accuracy for each trial block in Experiment 1 and pairwise block comparisons across the three study–test intervals (500 ms, 2000 ms, 5000 ms). The table reports t-values (*df* = 12, two-tailed p-values), Cohen’s d, and Bayes Factors (BF₁₀) for each comparison. These results provide the full statistical details underlying the significant pairwise effects summarized in the main text (Figure 2A).

| **Interval** | **Block**  **Comparison** | **Block 1**  **Mean(SD)** | **Block X Mean(SD)** | **t** | **p** | **Cohen's d** | **BF_10_** |
| --- | --- | --- | --- | --- | --- | --- | --- |
| 500 ms | 1 vs. 2 | 0.792(0.061) | 0.792(0.106) | 0 | 1 | 0 | 0.278 |
| 500 ms | 1 vs. 3 | 0.792(0.061) | 0.796(0.095) | 0.154 | 0.88 | 0.043 | 0.281 |
| 500 ms | 1 vs. 4 | 0.792(0.061) | 0.831(0.083) | 2.54 | 0.026 | 0.704 | 2.692 |
| 500 ms | 1 vs. 5 | 0.792(0.061) | 0.812(0.136) | 0.615 | 0.55 | 0.17 | 0.328 |
| 500 ms | 1 vs. 6 | 0.792(0.061) | 0.823(0.138) | 1 | 0.337 | 0.277 | 0.424 |
| 2000 ms | 1 vs. 2 | 0.742(0.106) | 0.777(0.115) | 1.354 | 0.201 | 0.376 | 0.589 |
| 2000 ms | 1 vs. 3 | 0.742(0.106) | 0.815(0.088) | 2.992 | 0.011 | 0.83 | 5.268 |
| 2000 ms | 1 vs. 4 | 0.742(0.106) | 0.823(0.122) | 2.54 | 0.026 | 0.705 | 2.694 |
| 2000 ms | 1 vs. 5 | 0.742(0.106) | 0.831(0.095) | 2.999 | 0.011 | 0.832 | 5.323 |
| 2000 ms | 1 vs. 6 | 0.742(0.106) | 0.873(0.101) | 3.187 | 0.008 | 0.884 | 7.082 |
| 5000 ms | 1 vs. 2 | 0.735(0.069) | 0.842(0.113) | 3.222 | 0.007 | 0.894 | 7.478 |
| 5000 ms | 1 vs. 3 | 0.735(0.069) | 0.900(0.119) | 4.116 | 0.001 | 1.141 | 29.209 |
| 5000 ms | 1 vs. 4 | 0.735(0.069) | 0.912(0.094) | 5.312 | <.001 | 1.473 | 169.051 |
| 5000 ms | 1 vs. 5 | 0.735(0.069) | 0.908(0.129) | 3.895 | 0.002 | 1.08 | 20.888 |
| 5000 ms | 1 vs. 6 | 0.735(0.069) | 0.923(0.099) | 5.696 | <.001 | 1.58 | 288.991 |
|  |  |  |  |  |  |  |  |

**Analysis of Experiment 1 data in 10-trial blocks**

In the main text, data for Experiment 1 were presented in blocks of 20 trials to allow direct comparison with previous relevant studies that also aggregated data in this way (Logie et al., 2009; Shimi & Logie, 2019). Also, participants were given a short rest in between each block of 20 trials. Olson and Jiang (2004) presented their data in three epochs (blocks) of 48 trials each.

Here we used a finer-grained analysis to present the results when the data were aggregated into blocks of 10 trials, resulting in 12 blocks for the 120 experimental trials. This approach allows a more fine-grained examination of performance changes over the course of the experiment, and is particularly relevant given our interest in when learning occurred across different study–test interval conditions.

A 12 (Block: 1–12) × 3 (Study–Test Interval: 500 ms, 2000 ms, 5000 ms) repeated-measures ANOVA on accuracy revealed a significant main effect of Block, *F*(11, 396) = 7.353, *p* < 0.001, *η_p_^2^* = 0.17, *BF_10_* > 1000 , and a non-significant main effect of Study-Test Interval, *F*(2, 36) = 2.57, *p* = .09, *η_p_^2^*= 0.13, *BF_10_* = 0.671. The Block × Interval interaction was significant, *F*(22, 396) = 2.10, *p* = 0.01, *η_p_^2^* = 0.11, *BF_10_* = 8.027. Pairwise comparisons indicated that for the 5000 ms study–test interval, performance began to improve from approximately Block 2 (Trials 11–20) onward; for the 2000 ms interval, stable improvement emerged from approximately Block 5 (Trials 41–50) onward; and for the 500 ms interval, no consistent improvement was observed across blocks.

The pattern of results is consistent with the main analysis based on 20-trial blocks: longer study–test intervals were associated with earlier and more pronounced performance improvements, while the shortest interval condition showed little or no improvement. The finer-grained 10-trial block analysis (**Figure S5**) also illustrates a gradual emergence of performance differences across intervals that becomes visible earlier in the experiment than in the coarser 20-trial block analysis, but still after multiple repetitions and not at all for the shortest study-test intervals.

**Figure S5.** Mean accuracy in Experiment 1 plotted by 10-trial blocks for each study–test interval condition. Error bars represent standard errors of the mean.

**Trial-type analyses for Experiment 1**

To examine whether the main effects observed in Experiment 1 were driven by participants' ability to detect a change on a trial-by-trial basis, we analysed the data separately for color change trials, shape change trials, and no-change trials. Results according to trial type are illustrated in **Figure S6**.

**Figure S6**. Mean percent accuracy for 500 ms, 2000 ms and 5000 ms across trial blocks for (a) color change trials, (b) shape change trials, and (c) no change trials in Experiment 1.

For color change trials (Figure S6a), we conducted a six (Block: 1–6) × three (Study–Test Interval: 500 ms, 2000 ms, 5000 ms) repeated-measures ANOVA. This revealed a significant main effect of Block, *F*(5, 180) = 8.93, *p* < 0.001, *η_p_^2^* = 0.20, *BF_10_* > 1000, indicating improved accuracy across Blocks. There was no significant main effect of Study–Test Interval, *F*(2, 36) = 0.03, *p* = .97, *η_p_^2^* = 0.00, *BF_10_* = 0.11, nor a significant Block × Interval interaction, *F*(10, 180) = 0.41, *p* = .91, *η_p_^2^* = 0.02, *BF_10_* = 0.02. Pairwise comparisons, shown in **Table S4**, revealed that detection of a color swap between shapes improved significantly in Blocks 3 to 6 versus with Block 1.

**Table S4**. Mean (SD) accuracy for color change trials across trial blocks, and pairwise block comparisons across study-test intervals (500 ms, 2000 ms, 5000 ms) in Experiment 1, along with t-value (*df* = 12, two-tailed p-value), *Cohen’s d*, and Bayes Factor (*BF₁₀*)

For shape change trials (Figure S6b), we conducted a six (Block: 1–6) × three (Study–Test Interval: 500 ms, 2000 ms, 5000 ms) repeated-measures ANOVA. This revealed a significant main effect of Block, *F*(5, 180) = 6.17, *p* < .001, *η_p_^2^* = 0.15, *BF_10_* = 483.85, indicating improved accuracy across Blocks. There was no significant main effect of Study–Test Interval, *F*(2, 36) = 1.58, *p* = .22, *η_p_^2^* = 0.08, *BF_10_* = 0.45. There was no significant Block × Interval interaction, *F*(10, 180) = 1.74, *p* = 0.09, *η_p_^2^* = 0.09, *BF_10_* = 0.55. Pairwise comparisons shown in **Table S5** revealed that detection of a shape swap between colors improved significantly in Blocks 2 to 6 versus Block 1.

**Table S5.** Mean (SD) accuracy for shape change trials across trial blocks, and pairwise block comparisons across study-test intervals (500 ms, 2000 ms, 5000 ms) in Experiment 1, along with t-value (*df* = 12, two-tailed p-value), *Cohen’s d*, and Bayes Factor (*BF₁₀*)

For no change trials (Figure S6c), we conducted a six (Block: 1–6) × 3 (Study–Test Interval: 500 ms, 2000 ms, 5000 ms) repeated-measures ANOVA. This revealed a non-significant effect of Study–Test Interval, *F*(2, 36) = 3.00, *p* = 0.06, *η_p_^2^* = 0.14, *BF_10_* = 1.27, no significant main effect of Block, *F*(5, 180) = 1.08, *p* =.37, *η_p_^2^* = 0.03, *BF_10_* = 0.06, and no significant Block × Interval interaction, *F*(10, 180) = 1.011, *p* = .43, *η_p_^2^* = 0.05, *BF_10_* = 0.09.

Results for analyses of trial types indicated that, across trials, participants gradually improved in their ability to detect a color change or shape change, and this pattern was the same regardless of the study-test interval. In contrast, for the trials on which no change occurred, performance did not improve across trials for any of the study-test intervals. Consistent with the main analysis, improvements on change trials occurred only after 60 trials for, on average 15 color change trials across the first three trial blocks, and after 40 trials for, on average 10 shape change trials across the first two trial blocks.

**Supplementary Analyses: Self-Reported Awareness**

In addition to the main analyses reported in the manuscript, we examined performance differences between participants who reported awareness of the repeated array and those who did not. The results of the mixed ANOVA are described in the main text (see Self-Reported Awareness of Repetition). Detailed pairwise comparisons between Block 1 and subsequent blocks for each awareness group are provided in **Table S6** below.

**Table S6.** Pairwise comparisons of mean accuracy (with SDs) between Block 1 and each subsequent block (Blocks 2–6) for the awareness (*df* = 14) and no-awareness (df = 23) groups in Experiment 1. The table reports t-values (*df* = 12, two-tailed p-values), *Cohen’s d*, and Bayes Factors (*BF₁₀*) for each comparison. These results provide the detailed statistical outcomes underlying the group differences summarized in the main text (Figure 2b).

| **Group** | **Block**  **Comparison** | **Block 1**  **Mean(SD)** | **Block X Mean(SD)** | **t** | **p** | **Cohen's d** | **BF_10_** |
| --- | --- | --- | --- | --- | --- | --- | --- |
| Awareness | 1 vs. 2 | 0.747(0.088) | 0.833(0.110) | 2.758 | 0.015 | 0.712 | 3.915 |
| Awareness | 1 vs. 3 | 0.747(0.088) | 0.913(0.074) | 5.169 | <.001 | 1.335 | 210.022 |
| Awareness | 1 vs. 4 | 0.747(0.088) | 0.900(0.098) | 4.258 | <.001 | 1.099 | 47.1 |
| Awareness | 1 vs. 5 | 0.747(0.088) | 0.947(0.064) | 7.135 | <.001 | 1.842 | 4067 |
| Awareness | 1 vs. 6 | 0.747(0.088) | 0.950(0.063) | 8.792 | <.001 | 2.27 | 36065 |
| No-awareness | 1 vs. 2 | 0.762(0.081) | 0.785(0.112) | 1.175 | 0.252 | 0.24 | 0.397 |
| No-awareness | 1 vs. 3 | 0.762(0.081) | 0.790(0.100) | 1.373 | 0.183 | 0.28 | 0.492 |
| No-awareness | 1 vs. 4 | 0.762(0.081) | 0.827(0.103) | 3.887 | <.001 | 0.793 | 45.429 |
| No-awareness | 1 vs. 5 | 0.762(0.081) | 0.790(0.116) | 1.143 | 0.265 | 0.233 | 0.384 |
| No-awareness | 1 vs. 6 | 0.762(0.081) | 0.825(0.121) | 2.186 | 0.039 | 0.446 | 1.576 |

**Pairwise Comparisons Across Blocks in Experiment 2**

To further explore the interaction between Block and Study–Test interval found in the accuracy analysis of Experiment 2, we conducted pairwise comparisons of accuracy across the six blocks within each Study–Test interval condition. This analysis was aimed at identifying whether performance changed systematically across blocks depending on the interval length.

The full results of all pairwise comparisons are presented in **Table S7**.

**Table S7.** Means (SDs) accuracy for each trial block, and pairwise block comparisons across study-test intervals (500 ms, 2000 ms, 5000 ms) in Experiment 2, along with t-value (*df* = 12, two-tailed p-value), *Cohen’s d*, and Bayes Factor (*BF_10_*). These results provide the detailed statistical outcomes underlying the group differences summarized in the main text (Figure 5a).

| **Interval** | **Block**  **Comparison** | **Block 1**  **Mean(SD)** | **Block X Mean(SD)** | **t** | **p** | **Cohen's d** | **BF_10_** |
| --- | --- | --- | --- | --- | --- | --- | --- |
| 500 ms | 1 vs. 2 | 0.815(0.092) | 0.838(0.065) | 0.64 | 0.534 | 0.177 | 0.332 |
| 500 ms | 1 vs. 3 | 0.815(0.092) | 0.762(0.106) | 1.923 | 0.079 | 0.533 | 1.147 |
| 500 ms | 1 vs. 4 | 0.815(0.092) | 0.827(0.090) | 0.288 | 0.778 | 0.08 | 0.289 |
| 500 ms | 1 vs. 5 | 0.815(0.092) | 0.769(0.115) | 1.075 | 0.303 | 0.298 | 0.452 |
| 500 ms | 1 vs. 6 | 0.815(0.092) | 0.769(0.088) | 1.315 | 0.213 | 0.365 | 0.566 |
| 2000 ms | 1 vs. 2 | 0.708(0.098) | 0.696(0.092) | 0.524 | 0.61 | 0.145 | 0.313 |
| 2000 ms | 1 vs. 3 | 0.708(0.098) | 0.704(0.085) | 0.173 | 0.866 | 0.048 | 0.282 |
| 2000 ms | 1 vs. 4 | 0.708(0.098) | 0.723(0.133) | 0.368 | 0.719 | 0.102 | 0.295 |
| 2000 ms | 1 vs. 5 | 0.708(0.098) | 0.788(0.102) | 2.114 | 0.056 | 0.586 | 1.478 |
| 2000 ms | 1 vs. 6 | 0.708(0.098) | 0.646(0.092) | 2.259 | 0.043 | 0.627 | 1.803 |
| 5000 ms | 1 vs. 2 | 0.692(0.086) | 0.658(0.098) | 0.845 | 0.415 | 0.234 | 0.377 |
| 5000 ms | 1 vs. 3 | 0.692(0.086) | 0.746(0.069) | 2.276 | 0.042 | 0.631 | 1.847 |
| 5000 ms | 1 vs. 4 | 0.692(0.086) | 0.650(0.122) | 0.882 | 0.395 | 0.245 | 0.387 |
| 5000 ms | 1 vs. 5 | 0.692(0.086) | 0.723(0.136) | 0.652 | 0.527 | 0.181 | 0.334 |
| 5000 ms | 1 vs. 6 | 0.692(0.086) | 0.688(0.121) | 0.09 | 0.929 | 0.025 | 0.279 |

**Accuracy by Trial Type in Experiment 2**

To further explore whether performance differed across trial types in Experiment 2, we conducted additional analyses by separating the data into color change, shape change, and no-change trials. We report the analysis of accuracy, and mean accuracy across blocks and trial types for study–test intervals: 500 ms vs. 2000 ms vs. 5000 ms, illustrated in **Figure S7**.

A series of six (Block: 1–6) × three (Study–Test Interval: 500 ms, 2000 ms, 5000 ms) repeated-measures ANOVAs were conducted separately for each trial type.

For color change trials, we conducted a 6 (Block: 1–6) × 3 (Study–Test Interval: 500 ms, 2000 ms, 5000 ms) repeated-measures ANOVA (**Figure S7a**). The analysis revealed no significant main effect of Block, *F*(5, 180) = 0.827, *p* = .515, *η_p_^2^* = 0.022, and no significant main effect of Study–Test Interval, *F*(2, 36) = 1.640, *p* = .208, *η_p_^2^* = 0.084. The interaction between Block and Study–Test Interval was also non-significant, *F*(10, 180) = 0.625, *p* = .765, *η_p_^2^* = 0.034. These results suggest that, for color change trials, accuracy remained stable across blocks and was not modulated by the length of the memory–test interval. Pairwise comparisons revealed that there was no significant difference between Block1 and Blocks 2 to 6.

For shape change trials, we conducted a 6 (Block: 1–6) × 3 (Study–Test Interval: 500 ms, 2000 ms, 5000 ms) repeated-measures ANOVA (**Figure S7b**). There was a significant main effect of Study–Test Interval, *F*(2, 36) = 3.923, *p* = .029, *η_p_^2^* = 0.179. However, no significant main effect of Block, F(5, 180) = 0.990, *p* = .418, *η_p_^2^* = 0.027, and no significant interaction between block and Study–Test Interval was found, *F*(10, 180) = 1.306, *p* = .230, *η_p_^2^* = 0.068. Pairwise comparisons revealed that there was no significant difference between Block1 and Blocks 2 to 6.

For no change trials, we conducted a 6 (Block: 1–6) × 3 (Study–Test Interval: 500 ms, 2000 ms, 5000 ms) repeated-measures ANOVA (**Figure S7c**). The analysis revealed a significant main effect of Study–Test Interval, *F*(2, 36) = 4.400, *p* = .020, *η_p_^2^* = 0.196, and a significant Block × Interval interaction, *F*(10, 180) = 2.071, *p* = .029, *η_p_^2^* = 0.103. However, there was no significant main effect of Block, *F*(5, 180) = 1.005, *p* = .410, *η_p_^2^* = 0.027. Pairwise comparisons revealed that there was no significant difference between Block1 and Blocks 2 to 6.

**Figure S7.** Mean percent accuracy for 500 ms, 2000 ms and 5000 ms across trial blocks for (a) color change trials, (b) shape change trials, and (c) no change trials in Experiment 2.

**Comparison of Experiments**

To further evaluate differences in performance between Experiment 1 and Experiment 2, we conducted additional pairwise comparisons across trial blocks. The results of the mixed-model ANOVA are described in the main text (see Comparison of Experiments). Detailed pairwise comparisons between the two experiments across Blocks 1–6 are presented in **Table S8** below.

**Table S8.** Pairwise comparisons of mean accuracy (with SDs) across Blocks 1–6 between participants in Experiment 1 and Experiment 2. The table reports t-values (*df* = 76, two-tailed p-values), Cohen’s d, and Bayes Factors (*BF₁₀*) for each comparison. These results provide the detailed statistical evidence for the differences between experiments summarized in the main text (Figure 5b).

| **Block** | **Exp. 1**  **Mean(SD)** | **Exp. 2**  **Mean(SD)** | **t** | **p** | **Cohen's d** | **BF_10_** |
| --- | --- | --- | --- | --- | --- | --- |
| 1 | 0.756(0.083) | 0.738(0.105) | 0.836 | 0.406 | 0.189 | 0.286 |
| 2 | 0.804(0.112) | 0.731(0.115) | 2.842 | 0.006 | 0.644 | 3.383 |
| 3 | 0.837(0.109) | 0.737(0.089) | 4.439 | <.001 | 1.005 | 93267 |
| 4 | 0.855(0.106) | 0.733(0.135) | 4.419 | <.001 | 1.001 | 50.623 |
| 5 | 0.850(0.125) | 0.760(0.119) | 3.249 | 0.002 | 0.736 | 28686 |
| 6 | 0.873(0.119) | 0.701(0.112) | 6.592 | <.001 | 1.493 | 89177 |

**References**

Logie, R.H., Brockmole, J.R. & Vandenbroucke, A. (2009). Bound feature combinations in visual short-term memory are fragile but influence long-term learning. *Visual Cognition,* 17, 160-179. https://doi.org/10.1080/13506280802228411

Olson, I. R., & Jiang, Y. (2004). Visual short-term memory is not improved by training. *Memory & Cognition*, 32(8), 1326–1332. <https://doi.org/10.3758/BF03206323>

Shimi, A., & Logie, R.H. (2019). Feature binding in short-term memory and long-term learning. *Quarterly Journal of Experimental Psychology*, 72, 1387-1400.

https:// doi.org//10.1177/1747021818807718
